# GhARF16‐1 modulates leaf development by transcriptionally regulating the *GhKNOX2‐1* gene in cotton

**DOI:** 10.1111/pbi.13484

**Published:** 2020-10-15

**Authors:** Peng He, Yuzhou Zhang, Hongbin Li, Xuan Fu, Haihong Shang, Changsong Zou, Jiří Friml, Guanghui Xiao

**Affiliations:** ^1^ College of Life Sciences Shaanxi Normal University Xi’an China; ^2^ Institute of Science and Technology Austria Klosterneuburg Austria; ^3^ College of Life Sciences Key Laboratory of Xinjiang Phytomedicine Resource and Utilization of Ministry of Education Shihezi University Shihezi China; ^4^ Zhengzhou Research Base State Key Laboratory of Cotton Biology Zhengzhou University Zhengzhou China; ^5^ Key Laboratory of Biological and Genetic Breeding of Cotton The Ministry of Agriculture Institute of Cotton Research Chinese Academy of Agricultural Sciences Anyang China; ^6^ Key Laboratory of Plant Stress Biology State Key Laboratory of Cotton Biology School of Life Sciences Henan University Kaifeng China

**Keywords:** leaf shape, auxin, cotton, transcriptional regulation, *GhARF16‐1*, *GhKNOX2‐1*

## Abstract

The leaf is a crucial organ evolved with remarkable morphological diversity to maximize plant photosynthesis. The leaf shape is a key trait that affects photosynthesis, flowering rates, disease resistance and yield. Although many genes regulating leaf development have been identified in the past years, the precise regulatory architecture underlying the generation of diverse leaf shapes remains to be elucidated. We used cotton as a reference model to probe the genetic framework underlying divergent leaf forms. Comparative transcriptome analysis revealed that the *GhARF16‐1* and *GhKNOX2‐1* genes might be potential regulators of leaf shape. We functionally characterized the auxin‐responsive factor ARF16‐1 acting upstream of *GhKNOX2‐1* to determine leaf morphology in cotton. The transcription of *GhARF16‐1* was significantly higher in lobed‐leaved cotton than in smooth‐leaved cotton. Furthermore, the overexpression of *GhARF16‐1* led to the up‐regulation of *GhKNOX2‐1* and resulted in more and deeper serrations in cotton leaves, similar to the leaf shape of cotton plants overexpressing *GhKNOX2‐1*. We found that GhARF16‐1 specifically bound to the promoter of *GhKNOX2‐1* to induce its expression. The heterologous expression of *GhARF16‐1* and *GhKNOX2‐1* in *Arabidopsis* led to lobed and curly leaves, and a genetic analysis revealed that *GhKNOX2‐1* is epistatic to *GhARF16‐1* in *Arabidopsis*, suggesting that the GhARF16‐1 and *GhKNOX2‐1* interaction paradigm also functions to regulate leaf shape in *Arabidopsis*. To our knowledge, our results uncover a novel mechanism by which auxin, through the key component ARF16‐1 and its downstream‐activated gene *KNOX2‐1*, determines leaf morphology in eudicots.

## Introduction

The aerial parts of higher plants are derived from a group of pluripotent stem cells in the shoot apical meristem (SAM), which leads to the generation of new outgrowth, leaves and flowers (Du *et al*., 2018; Shi *et al*., 2018). In comparison to the vibrant and diverse colours of flowers, leaves are typically more monotonously coloured. Nonetheless, leaves display remarkable morphological diversity with highly diversified geometry. Leaves can be simple with smooth margins or small marginal protrusions (serrations) as in *Arabidopsis thaliana*, while some can be complicated with protrusions of different sizes and patterns (e.g. teeth or lobes, depending on the depth of sinuses). Variation in leaf morphology is due to environmental or genetic factors (Nicotra *et al*., 2011). However, the extraordinary diversity is mostly attributed to genetic control, through which gene regulatory networks or signalling pathways control leaf formation from a small bulge on the SAM to a fully developed lateral outgrowth with diverse shapes (Dkhar and Pareek, 2014).

Genetic studies have identified the *KNOTTED1‐LIKE HOMEOBOX* (*KNOX*) family genes, such as *KNAT1*, *SHOOT MERISTEMLESS (STM)* and *BREVIPEDICELLUS* (*BP*), as key regulators of leaf shape (Chuck *et al*., 1996; Lincoln *et al*., 1994; Spinelli *et al*., 2011). In *Arabidopsis*, *KNAT2* is mainly expressed in the SAM and its overexpression induces highly lobed and curled rosette leaves in *Arabidopsis* (Chuck *et al*., 1996; Scofield *et al*., 2008). In *A. thaliana*, *ASYMMETRIC LEAVES1* (*AS1*) and *AS2* repress the activity of *KNAT1* and *KNAT2* in the leaves (Byrne *et al*., 2000; Byrne *et al*., 2002). Both *as1* and *as2* mutants display abnormal lobed leaves with ectopic expression of *BP* and *KNAT2* (Byrne *et al*., 2000; Byrne *et al*., 2002). A transcriptional repressor complex POLYCOMB REPRESSIVE COMPLEX (PRC)2 interacts with the AS1‐AS2 protein complex to stably silence the *KNAT1* and *KNAT2* genes in leaf primordia (Lodha *et al*., 2013). Nonetheless, it is still unknown how the key factor KNOX is enlisted to determine leaf diversity during the diversification of plant species.

In addition to the *KNOX* family genes, the *CUP‐SHAPED COTYLEDON* (*CUC*) and *REDUCED COMPLEXITY* (*RCO*) genes are also important regulators in determining leaf morphology (Bar and Ori, 2014; Bilsborough *et al*., 2011; Streubel *et al*., 2018; Vlad *et al*., 2014). *CUC2*, a member of the NAC gene family, plays a key role in the development of serrated leaf margins (Nikovics *et al*., 2006), and its ectopic expression leads to greater sinus width and lobe appearance rate in *Arabidopsis* (Blein *et al*., 2013). In *Cardamine hirsuta*, which belongs to the *Brassicaceae* family and shares a very close common ancestor with *Arabidopsis*, the homeodomain‐leucine zipper (HD‐ZIP) transcription factor gene *ChRCO* is specifically expressed in developing leaves and promotes leaflet development by repressing growth at the flanks of protrusions generated by CUC‐auxin patterning (Sicard *et al*., 2014; Vlad *et al*., 2014).

Moreover, auxin is involved in the regulation of leaf morphology. The local peaks of auxin activity are mediated by the auxin efflux carrier PIN‐FORMED. PIN1 regulates the growth of the leaf margin to affect leaf morphology (Barkoulas *et al*., 2008; Mohammed *et al*., 2018), which is evidenced by the fact that a loss‐of‐function mutation of *PIN1* leads to smooth margins in *C. hirsuta*. Auxin response factors (ARFs) regulate the expression of auxin‐responsive genes by directly binding to the auxin‐responsive element (AuxRE, TGTCTC) (Israeli *et al*., 2019). The ARF proteins contain two conserved domains, an N‐terminal DNA‐binding domain and a C‐terminal dimerization domain that mediates protein–protein interaction (Gray *et al*., 2001). In response to auxin, the auxin/indoleacetic acids (Aux/IAAs) are targeted for proteolysis through the ubiquitin‐mediated pathway, relieving ARFs, which regulate downstream gene transcription (Serino and Deng, 2003). Genetic analyses have shown that the *ARF10*, *ARF16* and *ARF17* genes regulate leaf morphology (Hendelman *et al*., 2012; Mallory *et al*., 2005; Wang *et al*., 2005). Interestingly, the *ARF10*, *ARF16* and *ARF17* mRNAs are targeted and negatively regulated by miR160 (Jones‐Rhoades *et al*., 2006; Natarajan and Banerjee, 2020; Sunkar *et al*., 2012). The expression of a miR160‐resistant form of *ARF10*/*ARF16*/*ARF17* results in the interference of auxin signalling, leading to the changes of leaf shape (Rubio‐Somoza *et al*., 2009). Likewise, in cotton, *ARF10*, *ARF16 and ARF17*, were also targeted by miR160. Overexpression of miR160 led to significant decrease of *ARF10* and *ARF17*, while down‐regulation of miR160 resulted in increased transcripts of *ARF10* and *ARF17* (Ding *et al*., 2017; Liu *et al*., 2020). Despite all these significant advances, the signalling pathways acting downstream of auxin to modulate leaf shape remain enigmatic. Furthermore, the genetic basis for the auxin‐mediated morphological diversity of leaves is still poorly understood.

In this study, we used *Gossypium* (cotton) species, one of the most important economic crops, to clarify the genetic underpinnings of the generation of divergent leaf forms. We show that the *Gossypium* auxin‐responsive factor ARF16‐1 acts as a key regulator that directly binds to the promoter of *KNOX2‐1* to fine‐tune its expression levels and, consequently, determines leaf shape diversity among the genus *Gossypium*. Notably, the *Gossypium* ARF16‐*KNOX2* interaction paradigm was also successfully implemented in *Arabidopsis,* which resulted in similar divergent leaf forms that were observed in *Gossypium* spp., suggesting that this regulatory mechanism is evolutionarily conserved in eudicots.

## Results

### Identification of candidate genes for divergent leaf shapes in *Gossypium* species

**Figure 1:**
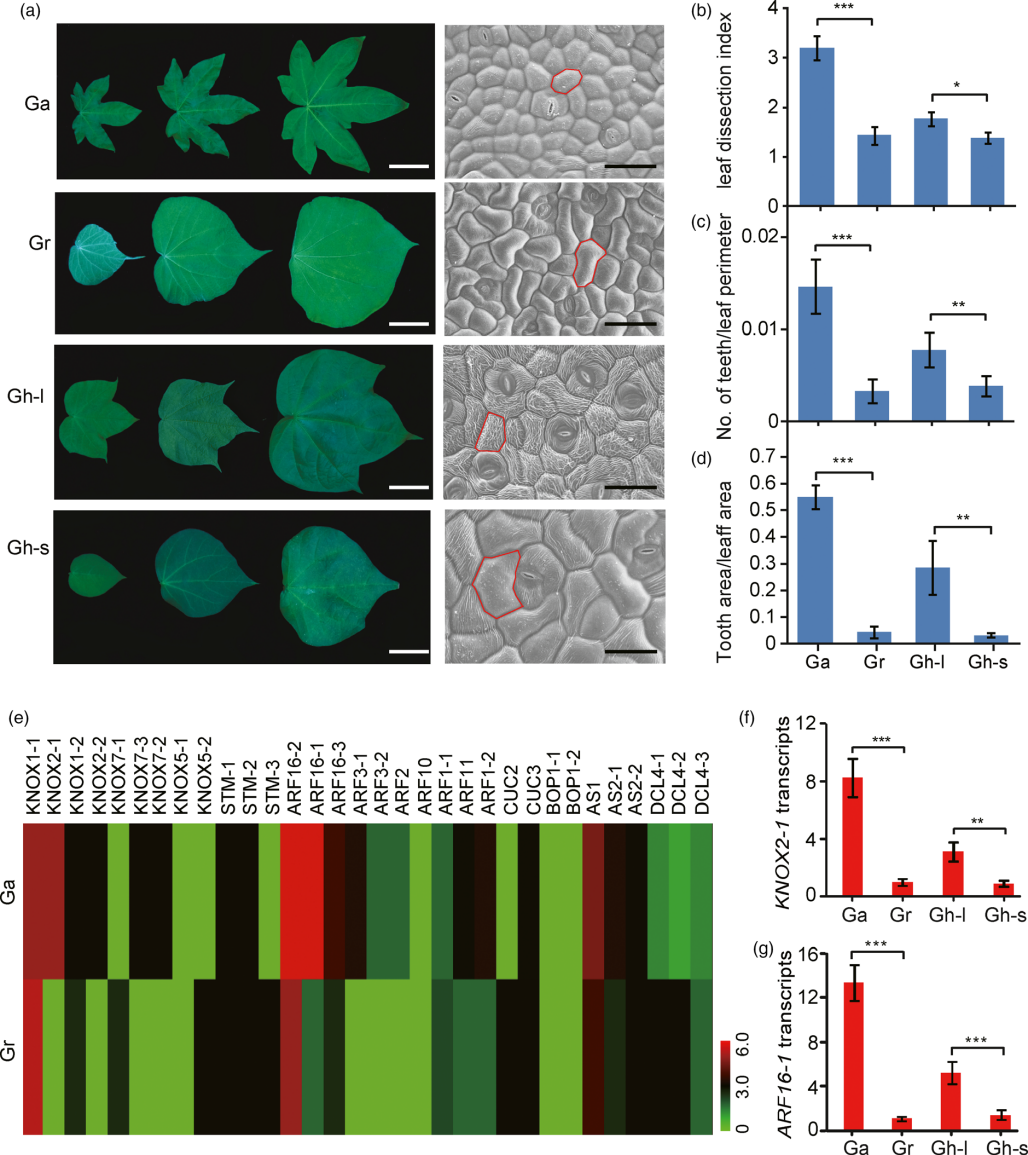
Candidate genes evolved to mediate leaf shape diversity among different *Gossypium* (cotton) species. (a) Left, morphological analysis of the leaves of *Gossypium hirsutum* (Gh), *Gossypium arboreum* (Ga), and *Gossypium raimondii* (Gr). From top to bottom, the first true leaf of *G. arboreum*, the first true leaf of *G. raimondii*, the fourth true leaf of *G. hirsutum* (Gh‐lobed, Gh‐l), and the first true leaf of *G. hirsutum* (Gh‐smooth Gh‐s). In each image, from left to right, the representative leaves from three different developmental stages (5, 15, and 30 days, respectively). Bars = 4 cm. The right column of the images shows scanning electron micrographs of the epidermal cells at the base of the abaxial side of mature leaves of Ga, Gr, Gh‐l, and Gh‐s, respectively, from top to bottom. Bars = 50 µm. (b) to (d) Quantitative comparisons of the leaf shapes of Ga, Gr, Gh‐l, and Gh‐s from (a) based on the leaf dissection index (perimeter^2^/4*π* × leaf area) (b), the number of teeth/leaf perimeter (c), and the tooth area/leaf area (d). A total of 10 leaves from each genotype were used for the measurement. Data are presented as mean ± SE. Error bars represent standard errors of the means from three independent experiments. Statistical significance was determined using one‐way analysis of variance (ANOVA) combined with Tukey's test: *P < 0.05, **P < 0.01, ***P < 0.001. (e) Expression profiles of the genes involved in the regulation of leaf shape in Ga and Gr. Scaled log2 expression values are shown from green to red, indicating low to high expression levels, respectively. (f) Quantitative real‐time PCR (qRT‐PCR) analysis of *KNOX2‐1* transcriptional levels in leaf primordia of Ga, Gr, Gh‐l, and Gh‐s. The expression level of the *KNOX2‐1* gene in Gr was set to 1.0. (g) qRT‐PCR analysis of the *ARF16‐1* mRNA levels in the leaf primordia of Ga, Gr, Gh‐l, and Gh‐s. The expression level of the *ARF16‐1* gene in Gr was set to 1.0. Each qRT‐PCR experiment was performed in three biological replicates, and the error bars represent standard errors of the means from three independent experiments. Statistical significance was determined using one‐way ANOVA combined with Tukey's test. **P < 0.01; ***P < 0.001.

*Gossypium* species exhibit diverse morphological variations because of natural selection and artificial domestication. Here, we used three completely sequenced cotton species, including one allotetraploid species, *G. hirsutum* and two diploid species, *G. arboreum* and *G. raimondii*, as reference plants to study leaf shape diversity as well as the genetic basis of the divergent leaf morphology during the diversification of the genus *Gossypium*. We showed that the leaves of *G. arboreum* were typically lobed, whereas those of *G. raimondii* were classic smooth (Figure 1a). Interestingly, we observed both lobed and smooth leaves occurring simultaneously in the same *G. hirsutum* plant. The shape of a leaf is determined by the geometric form of its epidermal cells (Tao *et al*., 2013). Therefore, we analysed the leaf epidermal cell shape using scanning electron microscopy. The epidermal cells of mature smooth leaves of *G. raimondii* and *G. hirsutum* showed a jigsaw‐puzzle‐piece shape, whereas the much smaller epidermal cells of mature lobed leaves of *G. arboreum* and *G. hirsutum* displayed a homogeneous shape. Furthermore, we used three different leaf parameters to quantitatively analyse the leaf shape of the three *Gossypium* species: (i) leaf dissection index (perimeter^2^/4*π* × leaf area), (ii) number of teeth/leaf perimeter, and (iii) tooth area/leaf area (Bilsborough *et al*., 2011; Royer *et al*., 2008; Zheng *et al*., 2016). The results showed that *G. arboreum* had a more pronounced leaf margin serration than *G. raimondii,* and distinct serrations were also observed between the lobed and smooth leaves of *G. hirsutum* (Figure 1b–d). Moreover, we calculated the ratio of lobed to smooth leaves in *G. hirsutum* and quantified the number of teeth on each leaf. Our calculations showed that the lobed leaves were the dominant type in *G. hirsutum* (Figure S1), and the number of teeth on each leaf in *G. raimondii* was nearly the same as that on a smooth leaf of *G. hirsutum*. However, the numbers of teeth on the lobed leaves of *G. arboreum* and *G. hirsutum* were much higher than those on the smooth leaves of *G. raimondii* and *G. hirsutum* (Figure S2). These results revealed the diversity in leaf margins of the three different cotton species.

To uncover the genetic underpinnings of leaf margin variation in *Gossypium*, we firstly analysed the differentially expressed genes between *G. arboreum* and *G. raimondii* using the RNA‐Seq data (Data S1). Subsequently, we summarized all genes potentially involved in the regulation of leaf morphological differences in *Arabidopsis* (Table S1). Finally, we investigated the expression of the cotton genes homologous to these potential *Arabidopsis* genes. Our results showed that the *KNOX2‐1* and *AUXIN RESPONSE FACTOR16* (*ARF16)* genes were significantly up‐regulated in the lobed leaves of *G. arboreum* compared to the smooth leaves of *G. raimondii* (Figure 1e), indicating that these genes might be recruited to regulate leaf shape differences among the *Gossypium* species. Moreover, the quantitative real‐time PCR (qRT‐PCR) results showed predominantly higher expression levels of *GhARF16‐1* and *GhKNOX2‐1* in lobed leaves than in smooth leaves, further supporting the expression profiles of *ARF16‐1* and *KNOX2‐1* (Figure S1f,g and S3). To confirm that dissimilar leaf profiles were attributed to the differentiated expression profiles of *ARF16‐1* and *KNOX2‐1*, we introduced another diploid *Gossypium* species, *G. trilobum*, whose genome constitution (D‐genome, DD) highly resembles that of *G. raimondii*. Compared to the smooth leaves of *G. raimondii*, the leaves of *G. trilobum* were lobed, as indicated by their greater values of leaf dissection index, number of teeth/leaf perimeter and tooth area/leaf area (Figure S4a–d). Consistent with the above‐mentioned phenotypic analysis results, the transcriptional levels of *ARF16‐1* and *KNOX2‐1* in *G. trilobum* leaves were much higher than those in *G. raimondii* leaves (Figure S4e,f), suggesting that *ARF16‐1* and *KNOX2‐1* might modulate leaf patterning during the diversification of these cotton species.

### *GhARF16‐1* and *GhKNOX2‐1* are key regulators that control cotton leaf shape

To verify that the two candidate genes, *GhARF16‐1* and *GhKNOX2‐1*, are the key regulators that modulate leaf shape variation in *Gossypium* species, we constructed several transgenic lines that overexpressed or down‐regulated *GhARF16‐1* and *GhKNOX2‐1* in *G. hirsutum*.

**Figure 2:**
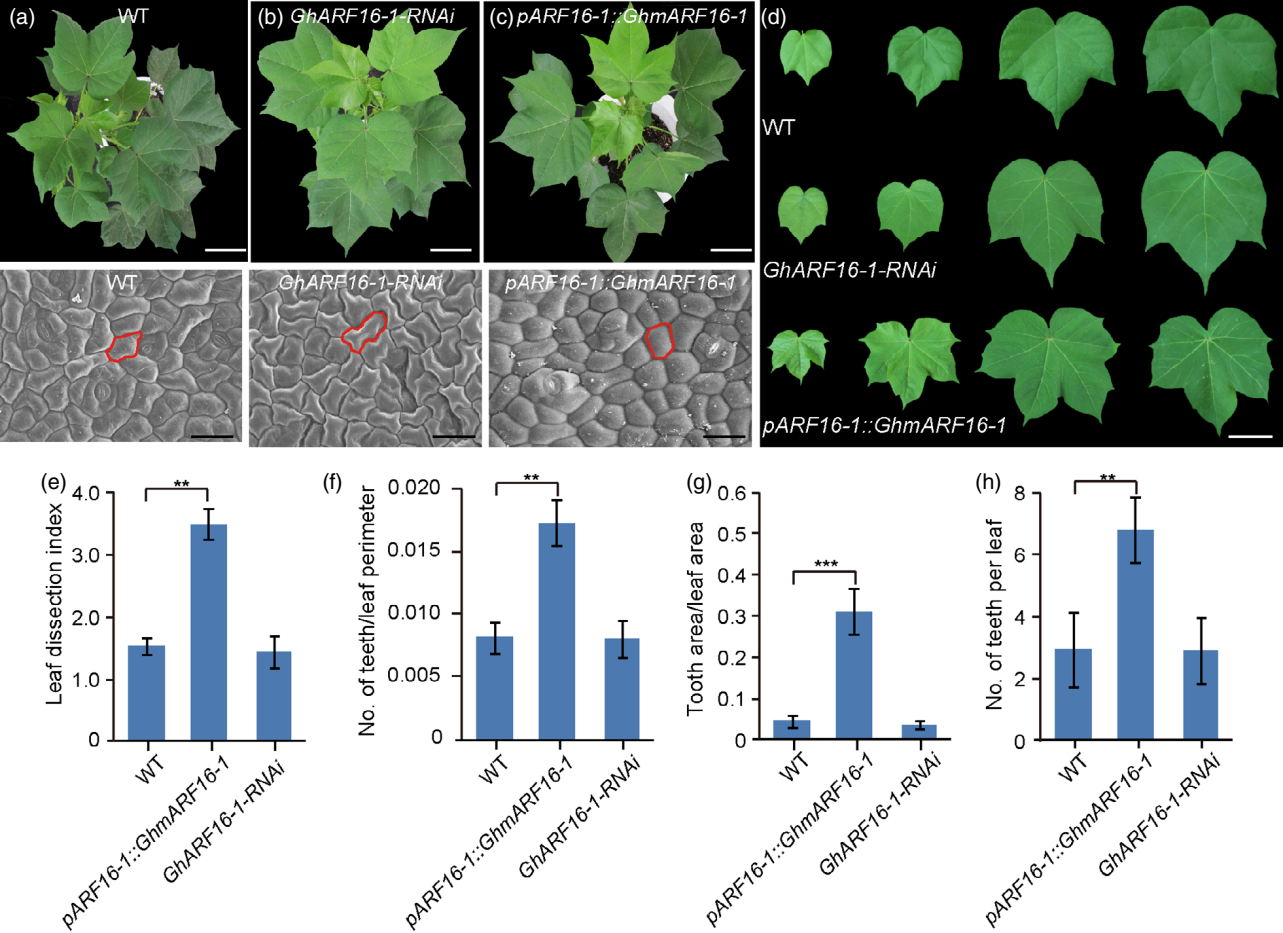
*GhARF16‐1* is involved in regulating leaf shape in cotton. (a)–(c) Top, from left to right, 48‐d‐old phenotypes of wild‐type *Gossypium hirsutum* (a), *GhARF16‐1* RNAi (b), and *pARF16‐1::GhmARF16‐1* transgenic *G. hirsutum* plants (c). Bars = 4 cm. Bottom, from left to right, scanning electron micrographs of the leaf epidermal cells at the base of the abaxial side of mature leaves of wild‐type, *GhARF16‐1* RNAi, and *pARF16‐1::GhmARF16‐1* transgenic lines. Bars = 50 µm. (d) Close‐up view of the leaves of 48‐d‐old wild‐type, *GhARF16‐1* RNAi, and *pARF16‐1::GhmARF16‐1* transgenic plants. (e)–(g) Quantitative comparisons of the leaf shapes of wild‐type, *GhARF16‐1* RNAi plants, and *pARF16‐1::GhmARF16‐1* transgenic lines based on the leaf dissection index (perimeter^2^/4*π* × leaf area) (e), the number of teeth/leaf perimeter (f), and the tooth area/leaf area (g). (h) Statistical analysis of number of teeth per leaf of wild‐type, *GhARF16‐1* RNAi, and *pARF16‐1::GhmARF16‐1* transgenic plants. A total of 10 leaves from each line were used for each measurement. Data are presented as mean ± SE. Statistical significance was determined using one‐way analysis of variance combined with Tukey's test. **P < 0.01; ***P < 0.001.

A sequence analysis of GhARF16‐1 showed that GhARF16‐1 had a high sequence similarity with *Arabidopsis* ARF16 (Figure S5), and *GhARF16‐1* mRNA containing an miR160 complementary sequence (Figure S6). To obtain a *GhARF16‐1* overexpressing line, a mutated version of *GhARF16‐1* (*GhmARF16‐1*), which modifies only miRNA160 complementary sequences by synonymous substitutions without altering the protein sequence (Figure S6) (Wang *et al*., 2005), was generated and introduced into cotton plants under the control of its own native promoter (Table S2). After pedigree selection assisted with kanamycin and transgene PCR amplification, 16 and 21 independent homozygous transgenic lines (approximately 10 plants per line) were regenerated for *pARF16::GhmARF16‐1* and *GhmARF16‐1‐RNAi*, respectively. Because the modification of miRNA160 disrupts its affinity for *GhmARF16‐1*, which results in the promotion of the stability of *GhmARF16‐1*, the expression level of *GhARF16‐1* in the *pARF16::GhmARF16‐1* lines was much higher than that in wild‐type *G. hirsutum* (Figure S7). As a result, these transgenic plants produced deeper lobed leaves as compared to the wild‐type leaves (Figure 2a,c,d). In contrast, the transgenic *GhARF16‐1*‐RNAi lines with reduced expression of *GhARF16‐1* exhibited leaf‐type similar to that of the wild type (Figure 2b,d). The epidermal cell shape was polygonal in mature leaves of wild‐type and *GhARF16‐1*‐RNAi lines, but was more regular in mature lobed leaves of *pARF16::GhmARF16‐1* (Figure 2a–c). The quantitative analysis of leaf morphology revealed that the *pARF16::GhmARF16‐1* plants had significantly greater leaf dissection index, number of teeth/leaf perimeter, tooth area/leaf area, and teeth number compared to the wild‐type and *GhARF16‐1*‐RNAi line (Figure 2e–h). These results suggest an important role of *GhARF16‐1* in regulating leaf shape in cotton.

**Figure 3:**
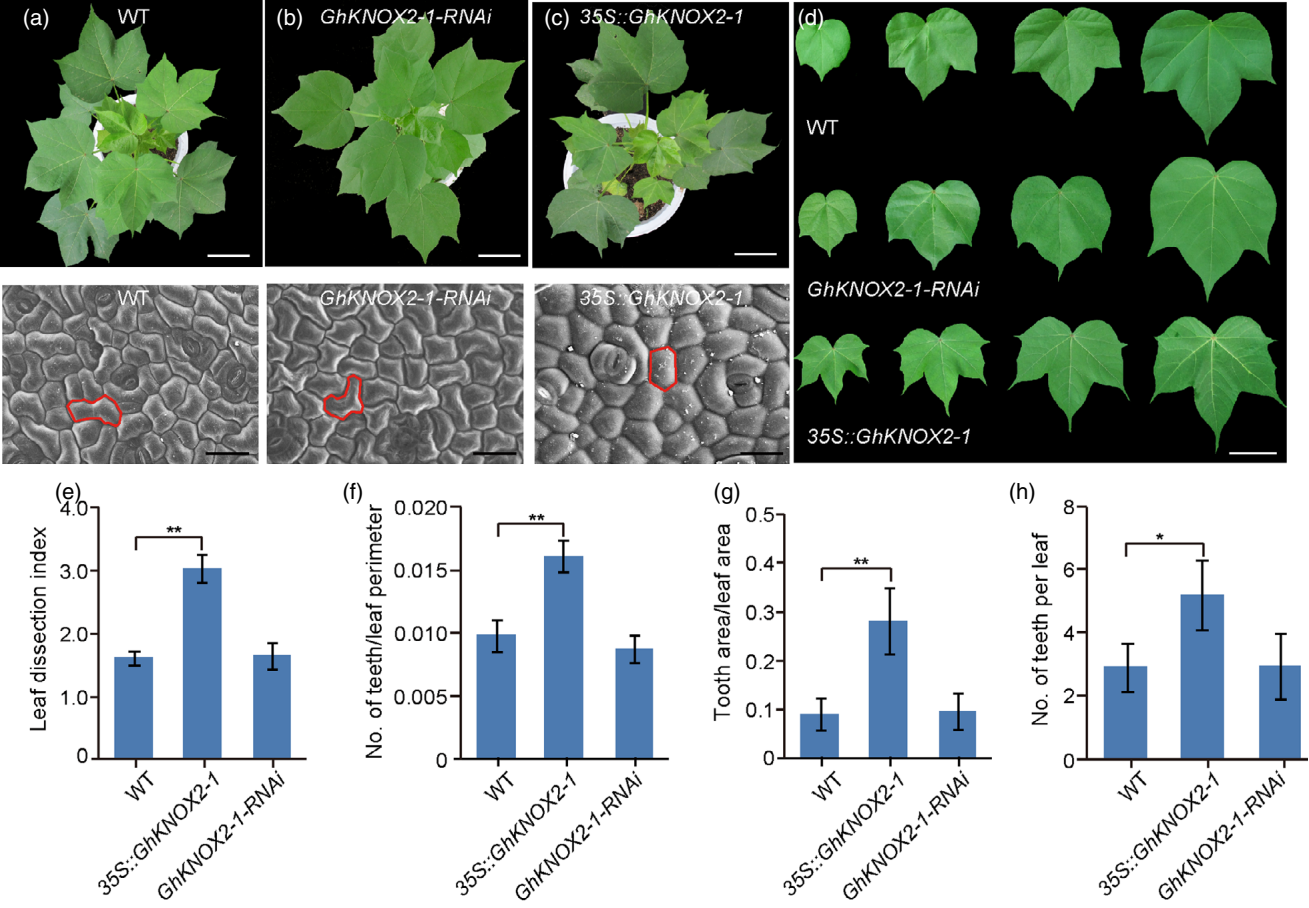
*GhKNOX2‐1* regulates leaf shape in cotton. (a)–(d) Top, from left to right, phenotypic analysis of 48‐d‐old wild‐type *Gossypium hirsutum* (a), *GhKNOX2‐1* RNAi (b), and *35S::GhKNOX2‐1* transgenic *G. hirsutum* plants (c). Bars = 4 cm. Bottom, from left to right, the scanning electron micrographs of the leaf epidermal cells at the base of the abaxial side of mature leaves of wild‐type, *GhKNOX2‐1* RNAi and *35S::GhKNOX2‐1* transgenic plants. Bars = 50 µm. (d) Close‐up images of the leaves of 48‐d‐old wild‐type plant (top), *GhKNOX2‐1* RNAi (middle) and 35S::*GhKNOX2‐1* transgenic plants (bottom). Bars = 4 cm. (e)–(g) Quantitative comparisons of leaf shapes of wild‐type, *GhKNOX2‐1* RNAi and *35S::GhKNOX2‐1* transgenic lines based on the leaf dissection index (perimeter^2^/4*π* × leaf area) (e), the number of teeth/ leaf perimeter (f) and the tooth area/leaf area (g). A total of 10 leaves from each line were used for each measurement. (h) Statistical analysis of number of teeth per leaf from the wild‐type, *GhKNOX2‐1* RNAi and *35S::GhKNOX2‐1* transgenic plants. A total of 10 leaves from each line were used for ach measurement. Data are presented as mean ± SE. Statistical significance was determined using one‐way analysis of variance combined with Tukey's test. *P < 0.05; **P < 0.01. WT, wild type.

To determine the role of *GhKNOX2‐1* in cotton leaf development, we generated the *GhKNOX2‐1* overexpression (*35S::GhKNOX2‐*1) and *GhKNOX2‐1* knocked down (*GhKNOX2‐1*‐RNAi) transgenic plants. After pedigree selection assisted with kanamycin and transgene PCR amplification, 34 and 16 independent homozygous transgenic lines (8–12 plants per line) were regenerated for *35S::GhKNOX2‐*1 and *GhKNOX2‐*1*‐RNAi*, respectively. The expression level of *GhKNOX2‐1* in the *35S::GhKNOX2‐1* line was much higher than that in the wild‐type plants (Figure S8). Compared with the wild‐type plants, the *35S::GhKNOX2‐1* plants displayed deeper lobed leaves (Figure 3a,c,d), resembling the leaves of *pARF16::GhmARF16‐1* (Figure 2a–d). Conversely, the *GhKNOX2‐1*‐RNAi line exhibited decreased *GhKNOX2‐1* transcription (Figure S8) and smooth leaves (Figure 3b,d), a phenotype similar to that of the wild type. Furthermore, the epidermal cell patterning of mature leaves of both wild‐type and *GhKNOX2‐1*‐RNAi lines were polygonal (Figure 3a,b), whereas the cells of mature leaves of the *35S::GhKNOX2‐1* line showed a more homogeneous shape (Figure 3c). Compared to the wild‐type plants, the *35S::GhKNOX2‐1* transgenic plants exhibited significantly greater values of the leaf dissection index, number of teeth/leaf perimeter, tooth area/leaf area and number of teeth on each leaf (Figure 3e,h). These results suggest that *GhKNOX2‐1* is also a key regulator that determines leaf shape in cotton.

### *GhKNOX2‐1* is the direct target of *GhARF16‐1*

**Figure 4:**
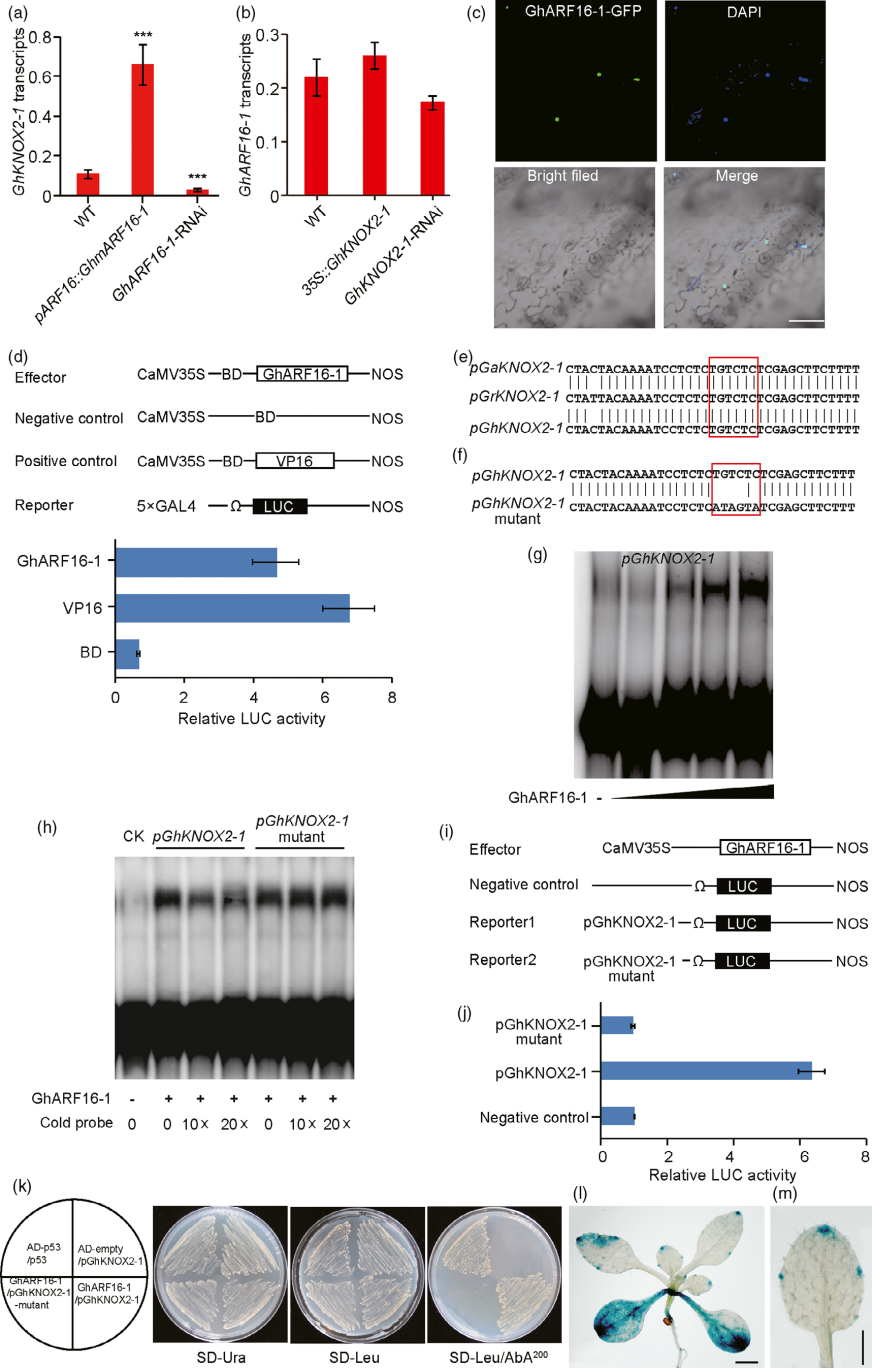
*GhKNOX2‐1* is targeted by GhARF16‐1. (a) qRT‐PCR analysis of the *GhKNOX2‐1* mRNA levels in wild‐type, *GhARF16‐1* RNAi and *pARF16::GhmARF16‐1* transgenic plants. (b) qRT‐PCR analysis of the *GhARF16‐1* mRNA levels in wild‐type, *GhKNOX2‐1* RNAi and *35S::GhKNOX2‐1* transgenic plants. Each qRT‐PCR experiment in (a) and (b) was performed in three biological replicates, and the error bars represent the standard errors of the means from three independent experiments. Statistical significance was determined using one‐way analysis of variance (ANOVA) combined with Tukey's test. ***P < 0.001. WT, wild type. (c) The subcellular localization of GhARF16‐1 in *Nicotiana benthamiana* leaf cells. Nuclei were counterstained with 4′,6′‐diamidino‐2‐phenylindole (DAPI). The merged images of green fluorescent protein (GFP), DAPI and bright‐field microscopy. (d) Transcriptional ability of GhARF16‐1 in *Arabidopsis* protoplasts. Upper panel, schematic diagrams of various constructs used in the transient expression assay. Lower panel, transcription activity of GhARF16‐1. The empty GAL4 DNA‐binding domain (BD) and BD‐VP16 vectors were used as negative and positive controls, respectively. The transcriptional assay was performed in three biological replicates, and the error bars represent the standard errors of the means from three independent experiments. (e) An ARF‐binding site was identified in the *KNOX2‐1* promoter region. The red rectangle indicates the predicted ARF‐binding sequence in *G. hirsutum* (Gh), *G. arboreum* (Ga) and *G. raimondii* Gr. (f) to (h) Electrophoretic mobility shift assay (EMSA) showed that GhARF16‐1 binds directly to the ARF‐binding site of the *GhKNOX2‐1* promoter. The ^32^P‐labelled DNA fragments of the *GhKNOX2‐1* promoter containing the intact (upper) or mutated (lower) ARF‐binding site (h) were incubated with the gradient concentrations of maltose‐binding protein (MBP)‐GhARF16‐1 fusion protein (g). The ^32^P‐labelled DNA fragments were incubated with MBP‐GhARF16‐1 to compete with different concentrations of the cold probes (without ^32^P‐labelling) of intact or mutated ARF‐binding site (h). (i) Schematic diagrams of various constructs used in the transient expression assay. (j) Transient expression assay of *GhKNOX2‐1* transcriptional activity modulated by GhARF16‐1 in *Arabidopsis* protoplasts. Either *pGhKNOX2‐1‐LUC* or *pGhKNOX2‐1 mutant‐LUC* was co‐transformed with the effector or empty vector (negative control) into *Arabidopsis* protoplasts. Transient expression assays were performed using three biological replicates, and the error bars represent the standard errors of the means from three independent experiments. (k) Yeast one‐hybrid assay of protein–DNA interaction. The 3x fragments of the *GhKNOX2‐1* promoter described in (e) and (f) were used. (l) and (m) GUS staining of the 25‐d‐old *Arabidopsis* seedlings expressing *pGhKNOX2‐1*:*:GUS*. Bars = 0.25 cm in (l) and 0.5 cm (m).

The lobed leaves of the *35S::GhKNOX2‐1* line are reminiscent of the leaf shape of the *pARF16::GhmARF16‐1* transgenic *G. hirsutum*, implying that *GhARF16‐1* and *GhKNOX2‐1* might regulate leaf shape in a common regulatory network. To preliminarily investigate a functional relationship between the *GhARF16‐1* and *GhKNOX2‐1* genes, we analysed the transcriptional level of *GhKNOX2‐1* in the *pARF16::GhmARF16‐1* and *GhARF16‐1*‐RNAi transgenic plants. The *GhKNOX2‐1* transcripts were up‐regulated in *G. hirsutum* plants expressing *GhmARF16‐1* and were markedly down‐regulated in the *GhARF16‐1*‐RNAi line compared to the wild‐type control plants (Figure 4a), suggesting that *GhKNOX2‐1* may act as a downstream target gene of GhARF16‐1. In line with this hypothesis, the transcriptional levels of *GhARF16‐1* in 35S::*GhKNOX2‐1* and *GhKNOX2‐1*‐RNAi transgenic plants were comparable to its transcriptional levels in the wild‐type plants (Figure 4b).

To investigate whether GhARF16‐1 and GhKNOX2‐1 are transcription factors, we analysed their subcellular localization. Our results showed that both GhARF16‐1 and GhKNOX2‐1 were localized to the nucleus (Figure 4c, Figure S9). A dual‐luciferase reporter assay was performed to further examine the transcriptional activity of GhARF16‐1 and GhKNOX2‐1 in *Arabidopsis* protoplasts. The results showed that the luciferase activities of GhARF16‐1 and GhKNOX2‐1 were much higher than those of the negative control (Figure 4d, Figure S10), indicating that both GhARF16‐1 and GhKNOX2‐1 have transcriptional ability *in vivo*.

Coincidently, the *cis*‐element analysis of the *GhKNOX2‐1* promoter (−1425 bp from the start codon ATG) showed that an AuxRE (TGTCTC) element was located at a region spanning from nucleotide −379 to −374 relative to the ATG start codon (Tables S3), which is a reported binding site for ARF transcription factor (Cherenkov *et al*., 2018). To confirm the interaction between the transcription factor GhARF16‐1 and the *GhKNOX2‐1* promoter, the *GhKNOX2‐1* promoter fragments containing either native or mutated ARF‐binding site were used separately for an electrophoretic mobility shift assay (EMSA) (Figure 4e,f). The results revealed that the recombinant GhARF16‐1 protein had significant affinity to the *GhKNOX2‐1* promoter fragment containing the AuxRE motif (Figure 4g). Moreover, the binding efficiency was gradually reduced by the addition of increasing amounts of unlabelled native fragment probes (cold probe) (Figure 4h). However, the application of the unlabelled mutated fragment probes did not affect the binding ability of GhARF16‐1 to the ^32^P‐label native *GhKNOX2‐1* promoter fragments. To further examine the DNA‐binding activity and transcriptional activity of GhARF16‐1 bound to the *GhKNOX2‐1* promoter, we performed a dual‐luciferase reporter assay, in which GhARF16‐1, driven by the *CaMV 35S* promoter, was coexpressed with a luciferase reporter driven by the *GhKNOX2‐1* promoter containing either a native or mutated ARF‐binding site (Figure 4i). Significant luciferase activity was detected only when GhARF16‐1 was coexpressed with the *GhKNOX2‐1* promoter containing the native ARF‐binding site (Figure 4j), suggesting that the AuxRE sequence in the *GhKNOX2‐1* promoter is essential for GhARF16‐1‐mediated transcriptional activation of *GhKNOX2‐1*. Additionally, a yeast one‐hybrid assay showed that GhARF16‐1 was able to bind to the *GhKNOX2‐1* promoter with the native but not the mutated AuxRE site (Figure 4k), which further confirmed the interplay between GhARF16‐1 and *GhKNOX2‐1* promoter *in vivo*.

Furthermore, to understand how the *GhKNOX2‐1* promoter regulates its gene expression in the leaf, a 1501‐bp *GhKNOX2‐1* promoter fragment upstream of the ATG initiation codon was fused with a β‐glucuronidase (*GUS*) reporter gene to generate the *pGhKNOX2‐1::GUS* construct. After transferring this construct into *Arabidopsis*, the expression patterns of the *GhKNOX2‐1* gene in *Arabidopsis* leaves were examined. Our results showed that a strong GUS activity was observed at the leaf margins (Figure 4l,m). Taken together, our results suggest that GhARF16‐1 binds to the AuxRE in the *GhKNOX2‐1* promoter and subsequently activates the expression of *GhKNOX2‐1* at the leaf margin, thus regulating leaf shape.

### GhARF16‐*GhKNOX2* signalling module regulates leaf shape in *Arabidopsis*

**Figure 5:**
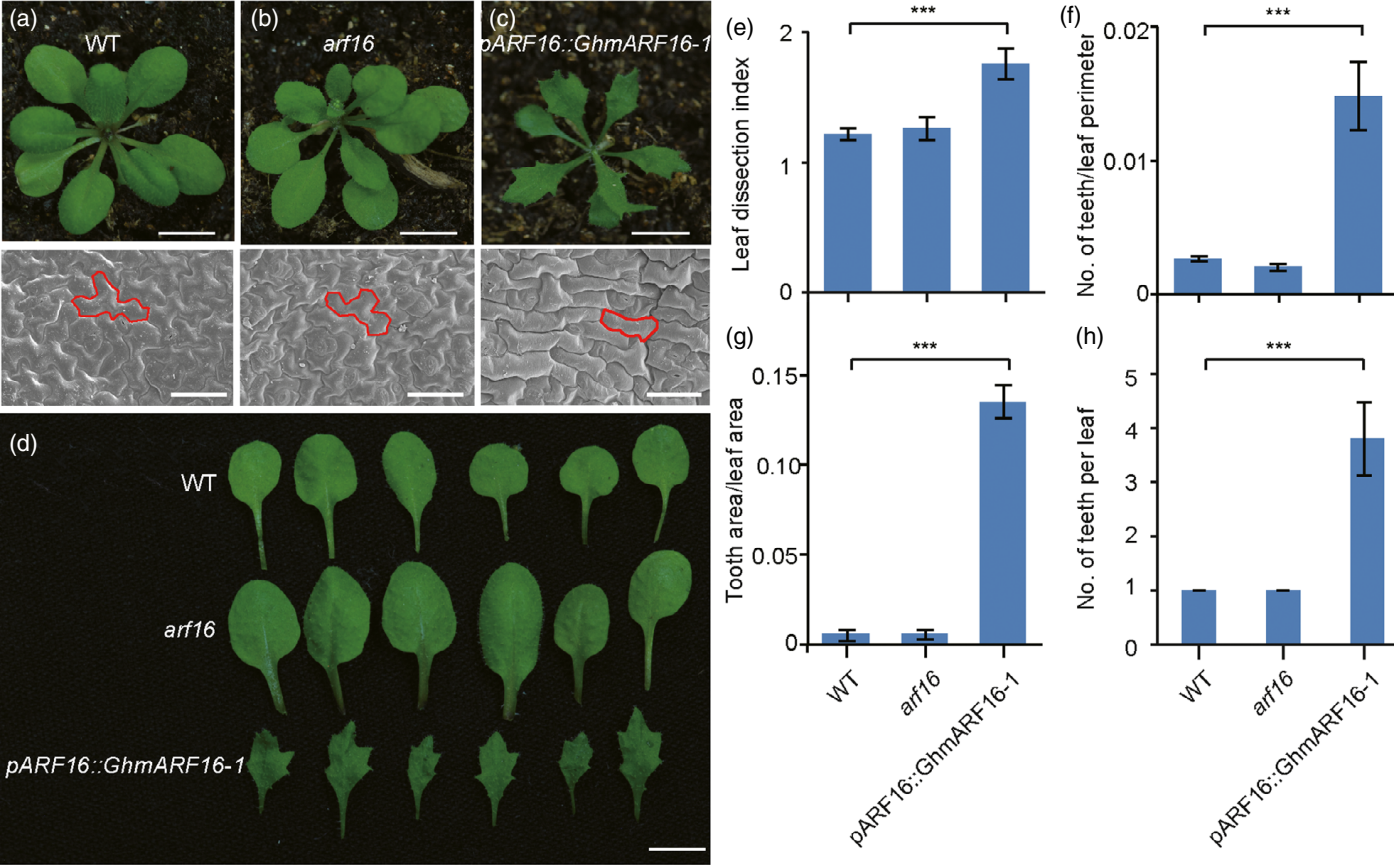
Cotton *GhARF16‐1* also functions in modulating leaf shape in *Arabidopsis thaliana*. (a)–(c) Top, from left to right, 21‐d‐old phenotypes of wild‐type (a), *arf16* mutant (b) and *pARF16::GhmARF16‐1* transgenic *Arabidopsis* plants (c). Bars = 4 mm. Bottom, from left to right, scanning electron micrographs of the leaf epidermal cells at the base of the abaxial side of mature leaves of wild‐type, *arf16* mutant and *pARF16::GhmARF16‐1* transgenic *Arabidopsis* plants. Bars = 50 µm. (d) Close‐up images of the leaves of 21‐d‐old wild‐type (top), *arf16* mutant (middle) and *pARF16::GhmARF16‐1* transgenic plants (bottom). (e)–(g) Quantitative comparisons of the leaf shapes of wild‐type, *arf16* mutant and *pARF16::GhmARF16‐1* transgenic plants based on the leaf dissection index (perimeter^2^/4*π* × leaf area) (e), number of teeth/leaf perimeter (f) and tooth area/leaf area (g). (h) Statistical analysis of the number of teeth per leaf from wild‐type, *arf16* mutant and *pARF16::GhmARF16‐1* transgenic plants. A total of 10 leaves from each line were used for each measurement. Data are presented as mean ± SE. Statistical significance was determined using one‐way analysis of variance combined with Tukey's test. ***P < 0.001. WT, wild type.

With respect to sequence similarity, the GhKNOX2‐1 and GhARF16‐1 proteins shared 71.12% and 62.14% identity with the AtKNAT2 and AtARF16 proteins, respectively. To clarify the GhARF16‐1‐mediated *GhKNOX2‐1* function in leaf morphology, we used the model flowering plant *Arabidopsis* to verify the working machinery. First, we expressed the gain‐of‐function *GhmARF16‐1* mutation into *Arabidopsis* under the control of its native promoter. A qRT‐PCR analysis revealed that the *GhARF16‐1* transcripts were strongly expressed in the *pARF16::GhmARF16‐1* transgenic *Arabidopsis* plants (Figure S11). Compared to the wild‐type and *arf16* mutant plants, the transgenic *Arabidopsis* plants carrying the *pGhARF16::GhmARF16‐1* construct displayed lobed leaves (Figure 5a–d, Figure S12), and their leaf epidermal cells were regular in shape. Moreover, the leaf patterning analysis indicated that the leaves of the *pGhARF16::GhmARF16* transgenic *Arabidopsis* plants displayed significantly higher leaf dissection index, number of teeth/leaf perimeter, tooth area/leaf area and teeth number (Figure 5e–h) than those of the wild‐type and *arf16* mutant plants. These results suggest that the heterologous expression of cotton *GhARF16* also promotes leaf serration in *Arabidopsis*. Notably, in *Arabidopsis*, expression of *mARF17* under its own promoter rather than *mARF16* phenocopied *pARF16::GhmARF16‐1* leaf shape, suggesting that cotton *GhARF16‐1* showed similar function to *Arabidopsis ARF17*. The reason might be that in *Arabidopsis*, *ARF16* and *ARF17* originated from a common ancestor gene, and slightly undergo the functional divergence during the evolution process. However, *ARF16* and *ARF17* genes are functional conserved in cotton, leading to the similar function between *GhARF16* and *Arabidopsis ARF17*.

**Figure 6:**
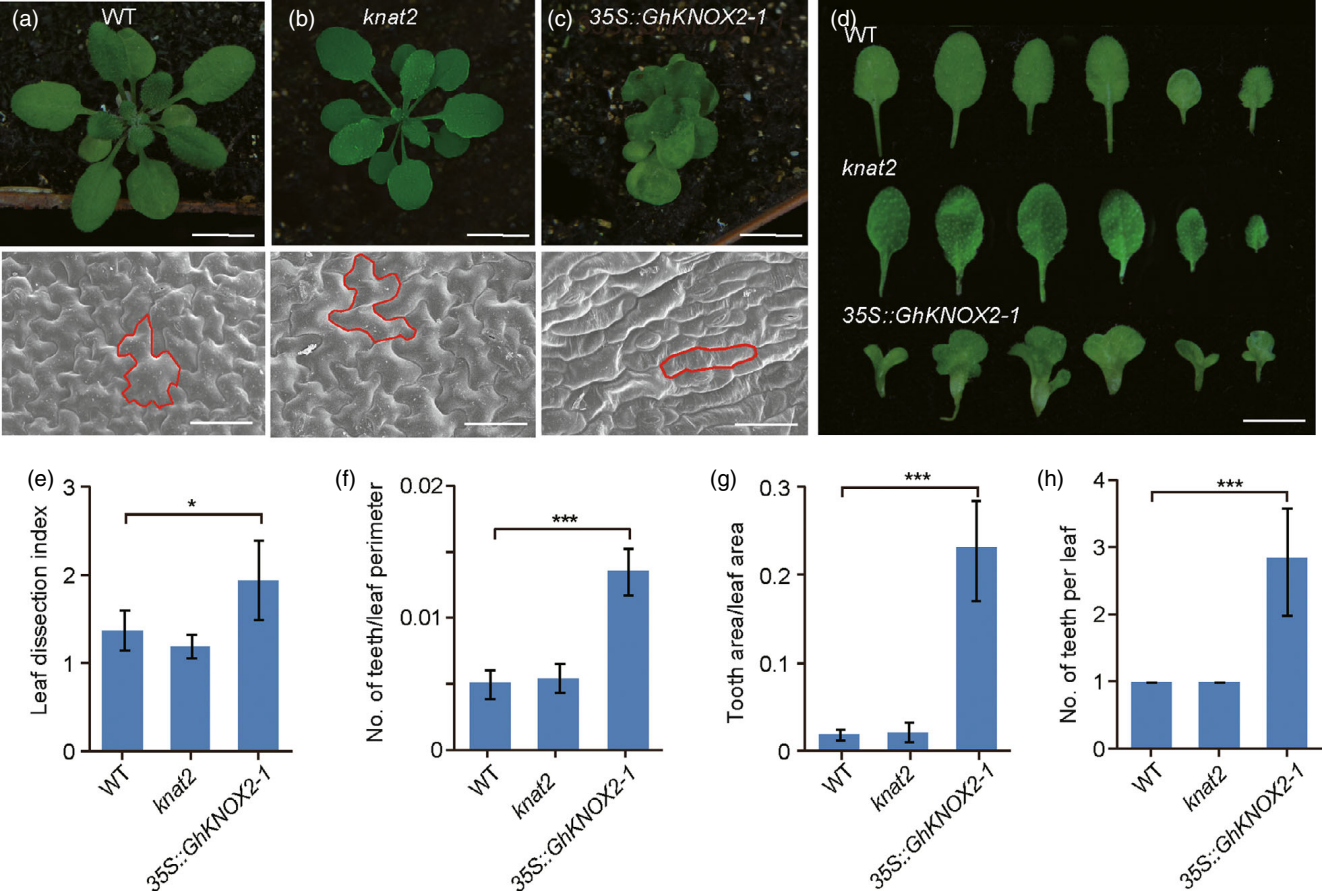
Ectopic expression of *GhKNOX2‐1* leads to lobed leaves in *Arabidopsis thaliana*. (a)–(d) Top, from left to right, 21‐d‐old phenotypes of wild‐type *A. thaliana* (a), *knat2* mutant (b) and *35S::GhKNOX2‐1* transgenic *Arabidopsis* (c). Bars = 4 mm. Bottom, from left to right, scanning electron micrographs of the leaf epidermal cells at the base of the abaxial side of mature leaves of wild‐type, *knat2* mutant and *35S::GhKNOX2‐1* transgenic *Arabidopsis*. Bars = 50 µm. (d) Close‐up images of the leaves of 21‐d‐old wild‐type (top), *knat2* mutant (middle) and *35S::GhKNOX2‐1* transgenic *Arabidopsis* (bottom). (e)–(g) Quantitative comparisons of the leaf shapes of wild‐type, *knat2* mutant and *35S::GhKNOX2‐1* transgenic lines based on the leaf dissection index (perimeter^2^/4*π* × leaf area) (e), number of teeth/leaf perimeter (f) and tooth area/leaf area (g). (h) Statistical analysis of the number of teeth per leaf of wild‐type, *knat2* mutant and *35S::GhKNOX2‐1* transgenic *Arabidopsis*. A total of 10 leaves of each phenotype were used for each measurement. Data are presented as the mean ± SE. Statistical significance was determined using one‐way analysis of variance combined with Tukey's test. *P < 0.05; ***P < 0.001. WT, wild type.

Likewise, we generated the *GhKNOX2‐1*‐overexpressing *Arabidopsis* transgenic line *35::GhKNOX2‐1*. A qRT‐PCR analysis revealed that *GhKNOX2‐1* was highly expressed in the *35::GhKNOX2‐1* transgenic *Arabidopsis* plants (Figure S13). Compared with the leaves of the wild‐type plants, the null mutant *knat2* exhibited a similar leaf phenotype with smooth margins, while the *35S::GhKNOX2‐1* line produced lobed leaves (Figure 6a–d, Figure S14). Similar to the observations in *Gossypium*, the epidermal cells of mature leaves of both wild‐type and *knat2* mutant *Arabidopsis* plants were polygonal, while the cells of mature lobed leaves of the *35S::GhKNOX2‐1* transgenic *Arabidopsis* plants showed a more homogeneous form. In contrast to the wild‐type leaves, the *35S::GhKNOX2‐1* transgenic *Arabidopsis* leaves showed greater values of leaf dissection index, number of teeth/leaf perimeter, tooth area/leaf area and number of teeth (Figure 6e–h). These results suggest that *GhKNOX2‐1*, which regulates *Gossypium* leaf patterning, can also function in the modification of *Arabidopsis* leaf shape.

### GhKNOX2‐1 functions epistatically to GhARF16‐1 to regulate leaf shape in Arabidopsis

**Figure 7:**
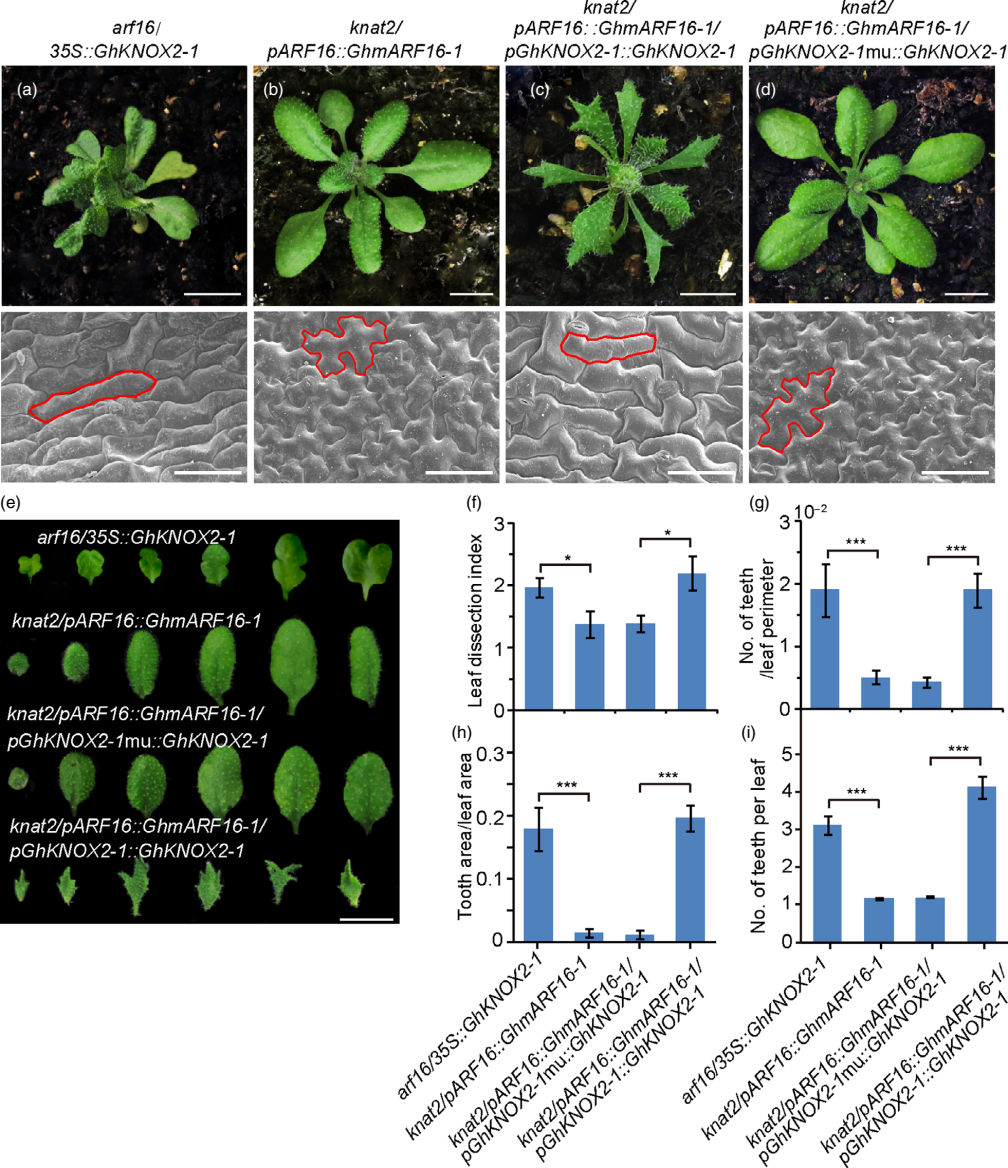
*GhKNOX2‐1* is functionally epistatic to *GhARF16‐1* in regulating leaf morphology. (a)–(d) Top, from left to right, 21‐d‐old phenotypes of the *arf16/35S::GhKNOX2‐1* transgenic *Arabidopsis* line overexpressing *GhKNOX2‐1* in the *arf16* mutant (a), *knat2/pARF16::GhmARF16‐1* line overexpressing *GhmARF16‐1* in the *knat2* mutant (b), *knat2/pARF16::GhmARF16‐1/pGhKNOX2‐1::GhKNOX2‐1* line overexpressing *GhARF16‐1* and *GhKNOX2‐*1 in the *knat2* mutant under the control of their respective native promoters (c) and *knat2/pARF16::GhmARF16‐1/pGhKNOX2‐1*mu*::GhKNOX2‐1* line overexpressing *GhARF16‐1* under the control of its native promoter and *GhKNOX2‐1* under the control of its mutated promoter (*pGhKNOX2‐1*mu; designated in Figure 4f) in the *knat2* mutant (d). Bars = 4 mm. Bottom, from left to right, scanning electron micrographs of the leaf epidermal cells at the base of the abaxial side of mature leaves of transgenic lines (a)–(d). Bars = 50 µm. (e) Close‐up images of the leaves of 21‐d‐old *Arabidopsis* transgenic lines (a)–(d). Bars = 4 mm. (f)–(h) Quantitative comparisons of the leaf shapes of (a)–(d) plants based on the leaf dissection index (perimeter^2^/4*π* × leaf area) (f), number of teeth/leaf perimeter (g) and tooth area/leaf area (h). (i) Statistical analysis of the number of teeth per leaf of transgenic plants (a)–(d). A total of 10 leaves from each phenotype were used for each measurement. Data are mean ± SE. Statistical significance was determined using one‐way analysis of variance combined with Tukey's test. *P < 0.05; ***P < 0.001.

Although the overexpression of the *G. hirsutum* genes *GhKNOX2‐1* and *GhARF16‐1* in *Arabidopsis* resulted in a phenotype similar to that in *Gossypium*, it is still unclear whether the GhARF16‐*GhKNOX2* interaction paradigm is also conserved in *Arabidopsis*. To elucidate this, we first generated the *arf16/35S::GhKNOX2‐1* transgenic *Arabidopsis* line overexpressing *GhKNOX2‐1* in the *arf16* mutant. As a result, the *arf16/35S::GhKNOX2‐1 Arabidopsis* produced irregularly shaped lobed leaves (Figure 7a,e), resembling the phenotype of the *35S::GhKNOX2‐1* line rather than the phenotype of the *arf16* mutant. In the transgenic *Arabidopsis* line *knat2*/*pARF16::GhmARF16‐1,* in which the gain‐of‐function *GhmARF16‐1* was heterologously expressed in the *Arabidopsis knat2* mutant under the control of its native promoter, the leaf patterning was similar to that shown by the *knat2* mutant with smooth and undivided margins, and its leaf epidermal cells were polygonal‐shaped. Overall, these characteristics of the transgenic line *knat2*/*pARF16::GhmARF16‐1* differed from those of *pARF16::GhmARF16‐1* (Figure 7b,e). These results suggest that *GhKNOX2‐1* can act as a downstream target of GhARF16‐1 to regulate leaf morphology in *Arabidopsis*.

To further investigate the crucial role of the AuxRE binding site in the *GhKNOX2‐1* promoter of the GhARF16‐*GhKNOX2* regulatory machinery, we expressed *GhKNOX2‐1* in *knat2/pARF16::GhmARF16‐1 Arabidopsis* using the native (*pGhKNOX2‐1*) or mutated (*pGhKNOX2‐1mu*) promoter (without AuxRE, Figure 4f). Compared to its strong expression in the *knat2/pGhKNOX2‐1::GhKNOX2‐1/pARF16::GhmARF16‐1* transgenic line with the native promoter *pGhKNOX2‐1*, the *GhKNOX2‐1* gene showed barely detectable transcription levels in the *knat2/pGhKNOX2‐1mu::GhKNOX2‐1/pARF16::GhmARF16‐1* transgenic *Arabidopsis* plants whose leaves displayed a smooth margin comparable to those of the *knat2* mutant (Figure 7c,d, Figure S15). An electron microscopic analysis revealed that the regular shape of epidermal cells was observed only in the lobed leaves of the transgenic line with the native promoter *pGhKNOX2‐1* (Figure 7c), but not in the line with the mutated promoter *pGhKNOX2‐1mu* (Figure 7d). Additionally, the *knat2/pGhKNOX2‐1::GhKNOX2‐1/pARF16::GhmARF16‐1* transgenic line carrying the native promoter *pGhKNOX2‐1* had significant positive effects on the leaf dissection index, number of teeth/leaf perimeter, number of teeth per leaf and tooth area/leaf area, compared to the *knat2/pGhKNOX2‐1mu::GhKNOX2‐1/pARF16::GhmARF16‐1* transgenic *Arabidopsis* with the mutated promoter *pGhKNOX2‐1mu* (Figure 7f–i) These results strongly suggest that GhARF16‐1 regulates *GhKNOX2‐1* via the AuxRE motif in the *GhKNOX2‐1* promoter, and the GhARF16‐*GhKNOX2* interaction paradigm can also regulate leaf shape in *Arabidopsis*.

## Discussion

**Figure 8:**
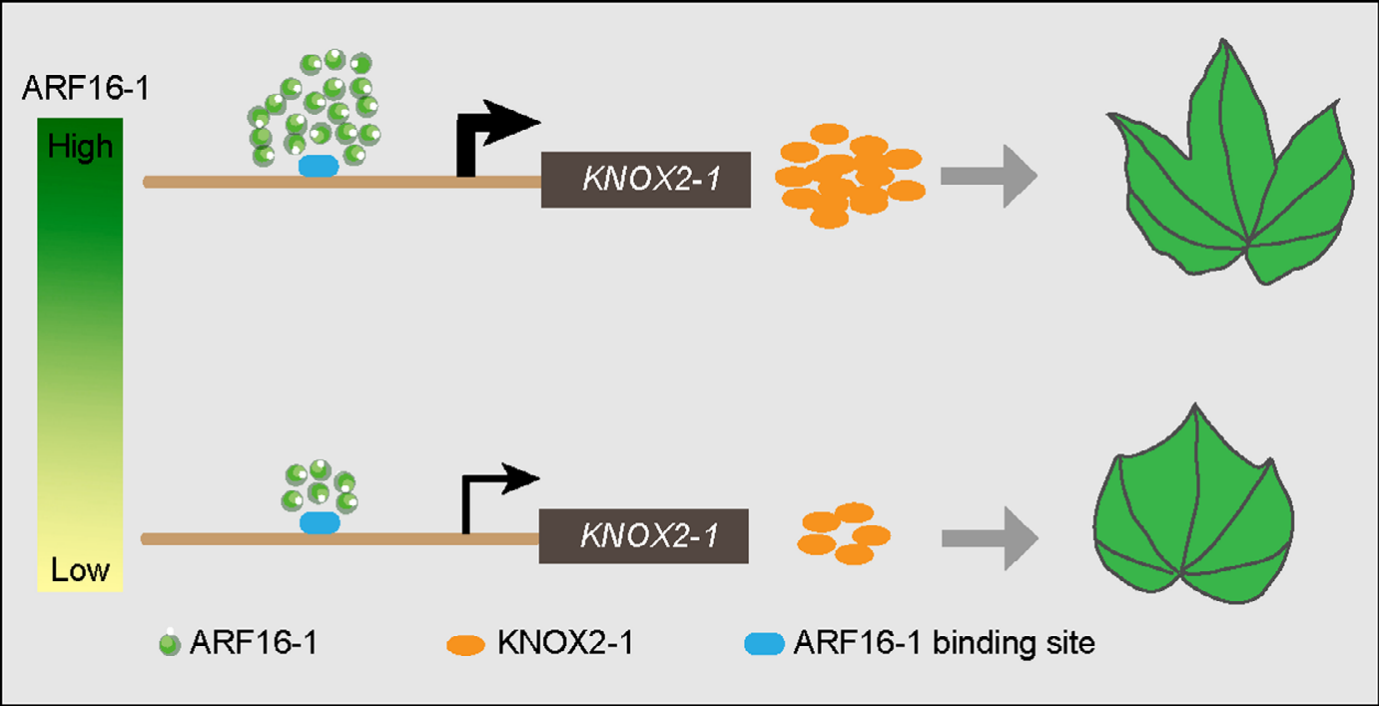
Working model for ARF16‐*KNOX2* interaction paradigm in regulating diverse leaf shapes. In the lobed leaves, where *ARF16‐1* is highly expressed, a large amount of ARF16‐1 protein binds directly to the ARF‐binding site [Auxin‐responsive element (AuxRE)] in the *KNOX2‐1* promoter regions to activate an abundance of *KNOX2‐1* transcripts that result in lobed leaves. In the smooth leaves, where *ARF16‐1* is weakly expressed, a deficiency of *GhARF16‐1* transcripts leads to a low expression level of *KNOX2‐1* that results in the production of smooth leaves.

Understanding the genetic basis of auxin signalling‐mediated leaf shape diversity provides an attractive opportunity to reveal the morphological diversity of a plant form during evolution. Recently, leaf morphological differences have been illustrated in numerous reference plants, including *Pelargonium* sp., grape vine (*Vitis* sp.), tomato (S*olanum lycopersicum*) and species of the poppy family (Papaveraceae), demonstrating that small genetic or hormonal changes can yield significantly different patterns (Bar and Ori, 2014; Scarpella *et al*., 2010; Scarpella *et al*., 2006). The divergent leaf shapes were also studied at the molecular level in a comparative context with two related plant species from the family *Brassicaceae*: *A. thaliana*, which has simple leaves with nearly smooth margins, and *C. hirsuta*, which has compound leaves with individual leaflets (Kierzkowski *et al*., 2019). Here, we used different species of the genus *Gossypium*, belonging to the mallow family *Malvaceae*, which is phylogenetically adjacent to the family *Brassicaceae*, as reference plants to illustrate differentiated leaf forms and reveal the genetic underpinnings of leaf shape diversity among *Gossypium* species. The *KNOX* family genes have been identified as the key regulators of leaf shape diversity in distinct plant species (Kierzkowski *et al*., 2019). Our work revealed that distinct amounts of ARF16‐1 proteins that bind to the AuxRE motif in the *KNOX2‐1* promoter can fine‐tune the expression levels of *KNOX2‐1* in different *Gossypium* species and, thus, determine their diverse leaf morphologies (Figure 8). Generally, this auxin signalling module mediating leaf shape might apply to other plant species in eudicots.

ARF transcription factors are able to be negatively regulated by different miRNAs. It has been reported that the *ARF10*, *ARF16* and *ARF17* were specifically targeted by miR160. ARF10, ARF16 and ARF17 shared very high sequence similarities and formed a subgroup (Figure S5) in the phylogenetic tree of ARF family members, suggesting that the miR160‐targeted ARFs are conserved for the ARF members in the same subgroup. In *Arabidopsis*, expression of a miR160‐resistant version of ARF17 under its native promoter resulted in rosette and cauline leaf margin serration (Mallory *et al*., 2005), whereas expression of a miR160‐resistant version of ARF16 resulted in upcurled and occasionally serrated leaves (Wang *et al*., 2005). Similarly, in cotton, *ARF10*, *ARF16 and ARF17* were also identified, and they were predicted to be targeted by miR160 (Liu *et al*., 2020). The previous work confirmed that overexpression of miR160 in cotton resulted in significant decrease of *ARF10* and *ARF17* (Ding *et al*., 2017). Our work further revealed that transgenic *Arabidopsis* carrying the *pGhARF16::GhmARF16‐1* construct displayed upcurled and occasionally serrated leaves (Figure 5), which phenotype was more similar to that in *Arabidopsis ARF16* overexpressing plants. Moreover, in tomato, constitutive expression of miR160‐resistant ARF10 caused a deeply divided leaf phenotype with completely separated stripe‐like lobes (Hendelman *et al*., 2012). In addition, overexpression of *miR160* gene under a constitutive *CaMV‐35S* promoter in tomato resulted in an entire leaflet blade phenotype compared to the wild‐type leaves. Meanwhile the expression level of the tomato Knotted 2 protein (Tkn2) was negatively correlated with miR160 levels and positively correlated with *ARF* gene expression levels, such as *ARF16* (Damodharan *et al*., 2018). Consistently, in our study, expression of a miR160‐resistant *GhARF16* gene in *Arabidopsis* and cotton led to significantly increased *GhKNOX2‐1* transcripts and deeply serrated leaf margins. We speculate that miR160 may cleave the *ARF16* transcripts and repress the expression of *ARF16*, which resulted in the down‐regulation of *KNOX2* gene. By elevating cytokinin levels and repressing gibberellin levels, which in turn promoted cell division and repressed cell differentiation, ARF‐*KNOX2* module participated in the establishment of leaf morphogenesis (Hay and Tsiantis, 2010; Jasinski *et al*., 2005; Yanai *et al*., 2005). Combing these studies, therefore we proposed that the miR160‐targeted ARF‐*KNOX2* module was conserved in leaf development.

In *A. thaliana* and *C. hirsuta*, researchers determined that two key genes, *STM* (KNOX family) and *RCO*, worked in combination to regulate variation in leaf shape (Hay and Tsiantis, 2010; Rast‐Somssich *et al*., 2015; Vlad *et al*., 2014; Vuolo *et al*., 2016). Here, we revealed that the *Gossypium* proteins KNOX2‐1 and ARF16‐1 likely act as two key regulators of leaf shape variation during the evolution or artificial domestication of *Gossypium* spp. By co‐manipulating *STM* (KNOX family member) and *RCO* in *Arabidopsis* leaves, Kierzkowski *et al*. (2019) produced a dissected leaf pattern resembling that of *C. hirsuta* (Kierzkowski *et al*., 2019). Likewise, by manipulating the expression of *GhKNOX2‐1* and *GhARF16‐1* in *G. hirsutum*, we were able to modify its original leaf form to resemble that of the other *Gossypium* species. Additionally, other KNOX or ARF members might be functionally redundant to the GhKNOX2 and GhARF16 proteins in the regulation of *Gossypium* leaf shape because the GhARF16‐1 and GhKNOX2‐1 RNAi transgenic lines showed no obvious alteration of leaf shapes.

Previous studies have indicated that the LATE MERISTEM IDENTITY 1 (LMI1)‐like transcription factor plays an important role in the regulation of cotton leaf shape (Andres *et al*., 2017; Chang *et al*., 2016). Silencing the *LMI1‐lik*e gene with the virus‐induced gene silencing (VIGS) method led to a change in cotton leaf patterning from okra to broad. The overexpression of the *LMI1‐like* gene significantly enhanced the depth of the leaf lobes in *Arabidopsis*. In *Arabidopsis*, *LMI1* was reported to directly activate *WEE1*, a conserved mitosis blocker, to regulate stipule proportions (Vuolo *et al*., 2018). Although the *LMI1‐like* gene was found to coordinate with the *KNOX* gene to regulate leaf development in plants (Chang *et al*., 2019), it remains to be investigated whether the Gossypium *LMI1‐like* gene is integrated into the GhARF16‐*GhKNOX2* network or it works in parallel with the ARF16‐*KNOX2* pathway to collaboratively regulate the complexities of leaf morphology in *Gossypium*.

The allotetraploid cotton, *G. hirsutum* (AADD genome), speciated through an allopolyploidization event in which an A‐genome species resembling *G. arboreum* (AA) and a D‐genome species resembling *G. raimondii* (DD) were reunited approximately 1 to 2 million years ago (Chen *et al*., 2020; Guan *et al*., 2014). We postulated that the *Gossypium* ARF16‐*KNOX2* interaction paradigm might have evolved prior to the formation of the allotetraploid cotton because the diploid cotton species *G. trilobum* (D‐genome) and *G. raimondii* (D‐genome) displayed divergent leaf shapes – lobed in *G. trilobum* and smooth in *G. raimondii* (Figure S4). Moreover, the differential expression levels of *ARF16‐1* and *KNOX2‐1* were also observed between the two diploid cotton species.

Our work not only provides new insights into the genetic basis of auxin‐regulated morphological diversity during plant evolution, but also offers a potential theoretical basis for breeding better varieties of crops. Leaf shape, one of most important characteristics of plants, directly affects crop growth and yield (Blein *et al*., 2008; Tsukaya, 2018). Leaf morphology can affect fibre yield, timing of crop maturation, disease resistance and even efficiency of foliar chemical application (Andres *et al*., 2014). Identifying genes involved in leaf shape and revealing the mechanisms of leaf morphogenesis will enrich our toolkit for agronomic trait improvement and horticultural applications in the future.

## Methods

### Plant materials and growth conditions

*Gossypium hirsutum* ‘Xuzhou 142,’ *G. arboreum* ‘Shixiya 1,’ *G. raimondii* ‘CMD10,’ and *G. trilobum* (Mocono & Sesse ex DC) were used in this study and grown in a climate‐controlled greenhouse with a 16‐h light and 8‐h dark cycle at 30 °C (Xiao *et al*., 2018). *Arabidopsis thaliana* plants were grown on soil in growth chambers with a 16‐h light and 8‐h dark cycle at 23 °C. The *Arabidopsis* mutant lines with disrupted *KNAT2* (*knat2‐5,* SALK_099837) and *ARF16* (*arf16‐2*, SALK_ 021448) were T‐DNA insertion alleles obtained from the SALK collections (*Arabidopsis* Biological Resource Center: Columbus, OH; http://signal.salk.edu). Seeds were surface sterilized with 0.1% HgCl_2_, germinated on Murashige and Skoog (MS) medium for 2 weeks, transferred to soil and grown in a fully automated control system (Percival) with a 16‐h light and 8‐h dark cycle at 23 °C in 70% humidity (Zhang *et al*., 2015).

### Cotton transformation

All overexpression and RNAi constructs were introduced into the *Agrobacterium* strain LBA4404. Seeds (*G. hirsutum* cv. YZ1) were sterilized and cultured in a chamber without light for 6 days at 30 °C. The *Agrobacterium*‐mediated cotton transformation was performed using hypocotyl segments as explants, as described previously (Jin *et al*., 2006; Li *et al*., 2009). After cutting into 5‐mm segments, the explants were immersed in the *Agrobacterium tumefaciens* suspension (OD = 0.2–0.4) for 15 min. The infected explants were transferred onto a callus‐induced medium. After callus induction, proliferation, embryogenic callus induction, embryo differentiation and plantlet regeneration, the putative transgenic plants were transferred into pots and grown in the greenhouse with a 14‐h light and 10‐h dark condition at 25 °C.

### RNA extraction and quantitative RT‐PCR (qRT‐PCR) analysis

For cotton samples, the plant material was frozen in liquid nitrogen and then ground to a fine powder with a mortar and pestle using a modified method (He *et al*., 2018). For *Arabidopsis* samples, total RNA was isolated from rosette leaves and seedlings at 0, 2, 4, 8, 12 and 20 days after germination. Total RNA was extracted using the PureLink™ RNA Mini Kit (Lot no. 1687455; Invitrogen: Waltham, MA) according to the manufacturer’s instructions, and cDNA was reverse‐transcribed from 5 μg of the total RNA as described previously (Xiao *et al*., 2018). The cDNA sequences of *G. KNOX* and *ARF* family genes were obtained from the Cotton Functional Genomics Database (https://cottonfgd.org/). The accession numbers and IDs of the identified genes are given in Tables S4 and S5. In the qRT‐PCR experiments, each gene was run in three biological and three technical replicates with the following reaction parameters: 95 °C for 10 min, followed by 40 cycles of 95 °C for 10 s and 56 °C for 30 s. A melting curve was generated from 65 to 95 °C. The SigmaStat software was used to statistically analyse data by one‐way analysis of variance (ANOVA). The housekeeping genes *GhUBQ7* and *AtUBQ5* were used as internal controls for cotton and *Arabidopsis*, respectively.

### RNA‐Seq analysis

The leaf primordia of *G. arboreum* and *G. raimondii* leaf were used for RNA extraction and RNA‐Seq. Total RNA was isolated using the PureLink^TM^ RNA Mini Kit (Invitrogen: Waltham, MA). The RNA was digested with RNase‐free DNase (Qiagen: Germantown, MD) and checked for integrity by capillary gel electrophoresis and Agilent 2100 Bioanalyzer (Agilent: Santa Clara, CA). The RNA [with RNA integrity number (RIN) ≥8] was pooled from the three biological replicates of each sample and then submitted to Novogene Bioinformatics Technology Co., Ltd. (Beijing, China) for high‐throughput sequencing via the Illumina HiSeq2000 sequencer. For RNA library construction, mRNA was enriched using oligo(dT) magnetic beads and then immediately broken into short segments in a fragmentation buffer. Subsequently, double‐stranded cDNA was synthesized and purified, followed by adaptor ligation. After the libraries were sequenced to generate > 30 million 125‐bp paired‐end (PE) reads for each sample, high‐quality reads were obtained after several steps of quality checks, which included trimming, removal of adaptor/primer sequences and low‐quality reads.

We used HISAT2 and Cufflinks software for the RNA‐seq expression analysis. In brief, RNA‐seq reads were mapped to the corresponding each cotton genome using HISAT2 (Version 2.1.0). Then, the expression of each gene was calculated using RPKM (Reads per kilobase of gene per million mapped reads) with Cufflinks (Version 2.2.1). The values for the heatmap in Figure 1e were displayed by log_2_RPKM. The differentially expressed genes between the two sets of samples were obtained by inputting read counts of each genes as expression matrix into the edgeR package. Only the genes with (*P* value <= 0.01 and fold change >= 2.0) were considered as significantly differential expressed.

### Transcription factor activity assay

The transcription factor assay was performed on *Arabidopsis* protoplasts (Wang *et al*., 2013). The reporter plasmid contained the firefly luciferase gene under the control of the 35S promoter with five GAL4‐binding elements upstream of the gene. The effector plasmids containing 35S‐BD and 35S‐BD‐VP16 were used as the negative and positive controls, respectively. The coding regions of *GhARF16‐1* and *GhKNOX2‐1* were amplified by PCR using the primers listed in Table S6, digested with Spe I and EcoR I, and cloned into the pRT‐BD vector to generate effector plasmids containing the 35S‐BD‐GhARF16‐1 and 35S‐BD‐GhKNOX2‐1 constructs, respectively. The reporter, effector and control plasmids were co‐transfected into *Arabidopsis* protoplasts by polyethylene glycol transformation. Luciferase assays were performed using the dual‐luciferase reporter assay system and a luminometer (GloMax20‐20, Promega: Madison, WI, USA).

### GUS staining and microscopy

Histochemical GUS staining was carried out as described previously (Tao *et al*., 2013). Tissues from the GhKNOX2‐1‐GUS transgenic lines were immersed in the staining buffer. Samples were vacuumed for 5 min and incubated overnight at 37 °C, after which the staining buffer was removed. Samples were stored in 70% ethanol before microscopic observation.

Scanning electron microscopy was conducted as described previously (Tao *et al*., 2013). The 5th leaf was sampled from the 25‐d‐old *Arabidopsis* plants and from 40‐d‐old cotton plants. The fifth‐leaf samples were fixed in FAA buffer (50% ethanol, 6% glacial acetic acid and 5% formaldehyde) for 4 h at 25 °C. After serial ethanol dehydration and isoamyl acetate substitution, the samples were dried in liquid carbon dioxide and then analysed using scanning electron microscopy (Hitachi S‐3400N, Hitachi, Tokyo, Japan).

### Transcriptional activation assay

The dual‐luciferase reporter assay was performed as described previously (Wang *et al*., 2013). The native coding region of *GhARF16‐1* was amplified by PCR using the primers listed in Table S6, digested with Spe I and EcoR I and cloned into the pRT‐BD vector driven by the constitutive 35S promoter to generate an effector plasmid. The native and mutated *GhKNOX2‐1* promoter sequences were separately amplified by PCR using the primers listed in Table S6, digested with HindIII and Pst I, and inserted into a pGreen‐LUC vector to drive the firefly luciferase reporter gene as reporter plasmids. The plasmid containing the Renilla luciferase gene, driven by the 35S promoter, was the control plasmid. The effector, reporter and internal control plasmids were mixed at a ratio of 6:6:1. These mixtures were then co‐transfected into *Arabidopsis* protoplasts by the polyethylene glycol transformation method (Zhang *et al*., 2015). After culturing for 16 h, the transcriptional activation assay was conducted by determining the luciferase activity using the dual‐luciferase reporter assay system according to the manufacturer's manual. The assay was performed with three biological replicates, and the error bars represent the standard errors of the means from three independent experiments.

### Electrophoretic mobility shift assay (EMSA)

The EMSA assay was performed using the chemiluminescent EMSA kit according to a previously described method (Hou *et al*., 2014). The promoter fragment of *GhKNOX2‐1* containing the native or mutant AuxRE element, was labelled with biotin on both ends of the probe. Sequences of DNA probes are as follows: Native probe, 5′–TAGTCATTAGCAGAGAGCATTAATTCTCACTTTCTATTACAAAATCCTCTCTGTCT CTCGAGCTTCTTTTGCACTTGTTTGGAAGAAAAGAAAGACAGAA‐3′, mutant probe, 5′‐TAGTCATTAGCAGAGAGCATTAATTCTCACTTTCTATTACAAAATCCTCTCATA GTATCGAGCTTCTTTTGCACTTGTTTGGAAGAAAAGAAAGACAGAA‐3′. The assay was conducted by incubating the labelled probe with purified GhARF16‐1 proteins at 25 °C for 1 h and separated by 8% native PAGE in 0.5X TBE buffer. Non‐labelled probes were used as cold competitors.

### Yeast one‐hybrid assay

The yeast one‐hybrid (Y1H) assay was performed as previously described (Xiao *et al*., 2018). Briefly, the ORFs of *GhARF16‐1* was constructed into the pGADT7 vector using homologous recombination. The 1425 bp sequence of *GhKNOX2‐1* promoter at the* *upstream of ATG (Tables S3) was amplified and inserted into the pAbAi vector with the Sac I and Kpn I. Y1HGold yeast strain cells were transformed with a pGADT7 prey vector carrying the ORF of *GhARF16‐1* and a constructed pAbAi bait vector described above. The transformed yeast cell suspension was dropped on SD [DDO for –Leu, with or without Aureobasidin A (AbA)] medium plates and cultured at 30 °C for 3 days.

### Leaf margin analysis

Leaf shape was recorded for transgenic cotton at the six‐leaf stage and then confirmed at the flowering stage. A leaf margin analysis was conducted as described previously (Zheng *et al*., 2016). Individual leaves were photographed using a camera (D3‐U3., Nikon, Tokyo, Japan). The leaf dissection index (perimeter^2^/4*π* × leaf area), number of teeth/leaf perimeter and/tooth area/leaf area were used to quantify leaf margins (Bilsborough *et al*., 2011). Leaf area and perimeter were measured using the ImageJ software. A total of 10 leaves each from wild‐type and mutant plants were chosen for analysis. Statistical significance was determined by one‐way ANOVA using the SigmaStat 3.5 software combined with Tukey's test.

### Vector construction and retro‐transformation

The full‐length sequences (from the start codon to the stop codon) of *GhKNOX2‐1* and *GhARF16‐1* were amplified from the cDNA using the primers listed in Table S6. For *GhmARF16‐1*, the miR160‐binding site of the *GhARF16‐1* gene was mutated to generate *GhmARF16‐1* according to a previous report (Wang *et al*., 2005). The product of each gene was digested with KpnI and XbaI, then cloned into the digested pQG110 vector driven by the constitutive cauliflower mosaic virus 35S promoter, and the construct was further introduced into *A. tumefaciens* strain GV3101 for the transformation of *A. thaliana* via the floral dip method. The transgenic plants were selected on solid half‐strength MS medium plates containing 50 mg/mL of appropriate antibiotics (Zhang *et al*., 2015) and were further validated by genomic PCR. The expression levels of *GhKNOX2‐1* and *GhARF16‐1* were also investigated in transgenic seedlings. Leaves from wild‐type and transgenic plants were used for further analysis.

## Conflict of interest

The authors declare that they have no conflict of interest.

## Author contributions

G.X. conceived the project and designed the experiments. P.H., Y.Z., X.F. and H.L. performed the experiments and analysed the data. H.S., C.Z. and J.F. wrote the manuscript with input from all authors.

## Supporting information


**Figure S1** Statistical analysis of the percentages of the lobed and smooth leaves in *Gossypium hirsutum*.
**Figure S2** Statistical analysis of the number of teeth per leaf from *Gossypium arboreum* (Ga), *G. raimondii* (Gr), Gh‐lobed (Gh‐l), and Gh‐smooth (Gh‐s).
**Figure S3** qRT‐PCR analysis of *KNOX1‐1*, *KNOX1‐2*, *KNOX2‐2*, *ARF16‐2,* and *ARF16‐3* mRNA levels from *Gossypium arboreum* (Ga), *G. raimondii* (Gr), Gh‐lobed (Gh‐l), and Gh‐smooth (Gh‐s).
**Figure S4** Analysis of leaf morphology and transcriptional levels of *KNOX2‐1* and *ARF16‐1* from *Gossypium raimondii* and *Gossypium trilobum*.
**Figure S4** A phylogenetic tree was constructed with MEGA 6.0 using the Maximum Likelihood (ML) method with 1000 bootstrap replicates based on a multiple alignment of the amino acid sequences of the *Arabidopsis* ARF proteins.
**Figure S5** Sequences of *GhARF16‐1*, *GhmARF16‐1*, and miR160.
**Figure S6** qRT‐PCR analysis of *GhARF16‐1* mRNA levels in wild‐type, *GhARF16‐1* RNAi, and *pARF16::GhmARF16‐1* transgenic plants.
**Figure S7** qRT‐PCR analysis of *GhKNOX2‐1* mRNA levels in wild‐type, *GhKNOX2‐1* RNAi, and *35S::GhKNOX2‐1* transgenic plants.
**Figure S8** Subcellular localization of GhKNOX2‐1 using *Nicotiana benthamiana* leaves.
**Figure S9** Transcriptional ability of GhKNOX2‐1 in *Arabidopsis* protoplasts.
**Figure S10** qRT‐PCR analysis of *GhARF16‐1* mRNA levels in *pARF16::GhmARF16‐1* transgenic *Arabidopsis*.
**Figure S11** Scanning electron micrographs of leaf epidermal cells at the base of the abaxial side of mature leaves of wild‐type, *arf16* mutant, and *pARF16::GhmARF16‐1* transgenic plants.
**Figure S12** qRT‐PCR analysis of *GhKNOX2‐1* mRNA levels in *35::GhKNOX2‐1* transgenic *Arabidopsis*.
**Figure S13** Scanning electron micrographs of leaf epidermal cells at the base of the abaxial sides of mature leaves of wild‐type, *knat2* mutant, and *35S::GhKNOX2‐1* transgenic plants.
**Figure S14** qRT‐PCR analysis of *GhKNOX2‐1* mRNA levels in leaves from individual lines shown in Figure 5A to 5D.
**Table S1** List of genes related to leaf shape development in *Arabidopsis*.
**Table S2** Promoter sequence of *GhARF16‐1*.
**Table S3.** Promoter sequence of *GhKNOX2‐1*.
**Table S4** Analysis of *Gossypium hirsutum* *KNOX* gene family and its orthologs in AA and DD cotton genomes.
**Table S5** Analysis of *Gossypium hirsutum* *ARF* gene family and its orthologs in AA and DD cotton genomes.
**Table S6** List of primers used in this study.

**Data S1** Differentially expressed genes between *Gossypium arboreum* and *Gossypium raimondi*.
