# Supplementary material for: GhARF16‐1 modulates leaf development by transcriptionally regulating the GhKNOX2‐1 gene in cotton
Source: Plant Biotechnol J. 2020 Oct 15;19(3):548–62. doi: 10.1111/pbi.13484 (PMC7955886; doi:10.1111/pbi.13484)
Supplement: Supplementary file 1 — Figure S1 Statistical analysis of the percentages of the lobed and smooth leaves in Gossypium hirsutum. Figure S2 Statistical analysis of the number of teeth per leaf from Gossypium arboreum (Ga), G. raimondii (Gr), Gh‐lobed (Gh‐l), and Gh‐smooth (Gh‐s). Figure S3 qRT‐PCR analysis of KNOX1‐1, KNOX1‐2, KNOX2‐2, ARF16‐2, and ARF16‐3 mRNA levels from Gossypium arboreum (Ga), G. raimondii (Gr), Gh‐lobed (Gh‐l), and Gh‐smooth (Gh‐s). Figure S4 Analysis of leaf morphology and transcriptional levels of KNOX2‐1 and ARF16‐1 from Gossypium raimondii and Gossypium trilobum. Figure S4 A phylogenetic tree was constructed with MEGA 6.0 using the Maximum Likelihood (ML) method with 1000 bootstrap replicates based on a multiple alignment of the amino acid sequences of the Arabidopsis ARF proteins. Figure S5 Sequences of GhARF16‐1, GhmARF16‐1, and miR160. Figure S6 qRT‐PCR analysis of GhARF16‐1 mRNA levels in wild‐type, GhARF16‐1 RNAi, and pARF16::GhmARF16‐1 transgenic plants. Figure S7 qRT‐PCR analysis of GhKNOX2‐1 mRNA levels in wild‐type, GhKNOX2‐1 RNAi, and 35S::GhKNOX2‐1 transgenic plants. Figure S8 Subcellular localization of GhKNOX2‐1 using Nicotiana benthamiana leaves. Figure S9 Transcriptional ability of GhKNOX2‐1 in Arabidopsis protoplasts. Figure S10 qRT‐PCR analysis of GhARF16‐1 mRNA levels in pARF16::GhmARF16‐1 transgenic Arabidopsis. Figure S11 Scanning electron micrographs of leaf epidermal cells at the base of the abaxial side of mature leaves of wild‐type, arf16 mutant, and pARF16::GhmARF16‐1 transgenic plants. Figure S12 qRT‐PCR analysis of GhKNOX2‐1 mRNA levels in 35::GhKNOX2‐1 transgenic Arabidopsis. Figure S13 Scanning electron micrographs of leaf epidermal cells at the base of the abaxial sides of mature leaves of wild‐type, knat2 mutant, and 35S::GhKNOX2‐1 transgenic plants. Figure S14 qRT‐PCR analysis of GhKNOX2‐1 mRNA levels in leaves from individual lines shown in Figure 5A to 5D. Table S1 List of genes related to leaf shape development in Arabidopsis. Tabl [file PBI-19-548-s001.docx]

**Supporting Information**

**Supplemental Figure S1** Statistical Analysis of the Percentages of the Lobed and Smooth Leaves in *G. hirsutum*. Gh-l and Gh-s Represent *G. hirsutum* Lobed Leaves and *G. hirsutum* Smooth Leaves.

**
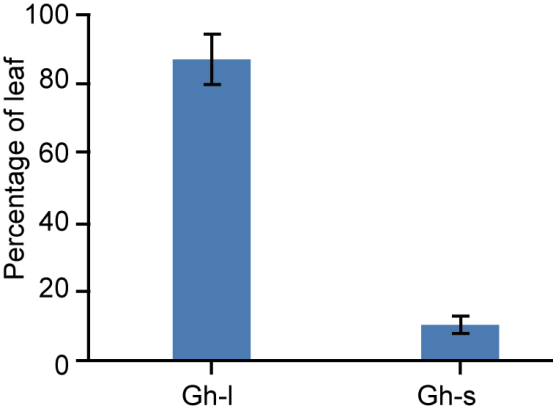
**

**Supplemental Figure S2** Statistical Analysis of the Number of Teeth per Leaf from Ga, Gr, Gh-l and Gh-s. Statistical significance was determined using one-way analysis of variance combined with Tukey’s test. **, *P<*0.01; ***, *P<*0.001. Ga, Gr, Gh-l and Gh-s represent *G. arboreum* leaves, *G. raimondii* leaves, *G. hirsutum* lobed leaves and *G. hirsutum* smooth leaves.

**
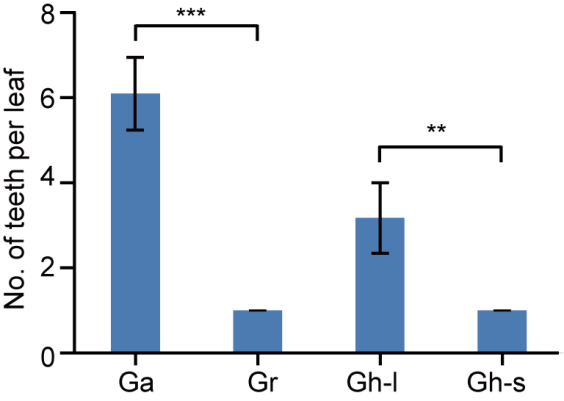
**

**Supplemental Figure S3** qRT-PCR Analysis of *KNOX1-1*, *KNOX1-2*, *KNOX2-2*, *ARF16-2* and *ARF16-3* mRNA Levels from Ga, Gr, Gh-l and Gh-s. The expression level of each gene in Gr was set to 1.0. Each qRT-PCR experiment was performed in three biological replicates, and the error bars represent standard errors of the means from three independent experiments. Ga, Gr, Gh-l and Gh-s represent *G. arboreum* leaves, *G. raimondii* leaves, *G. hirsutum* lobed leaves and *G. hirsutum* smooth leaves.

**
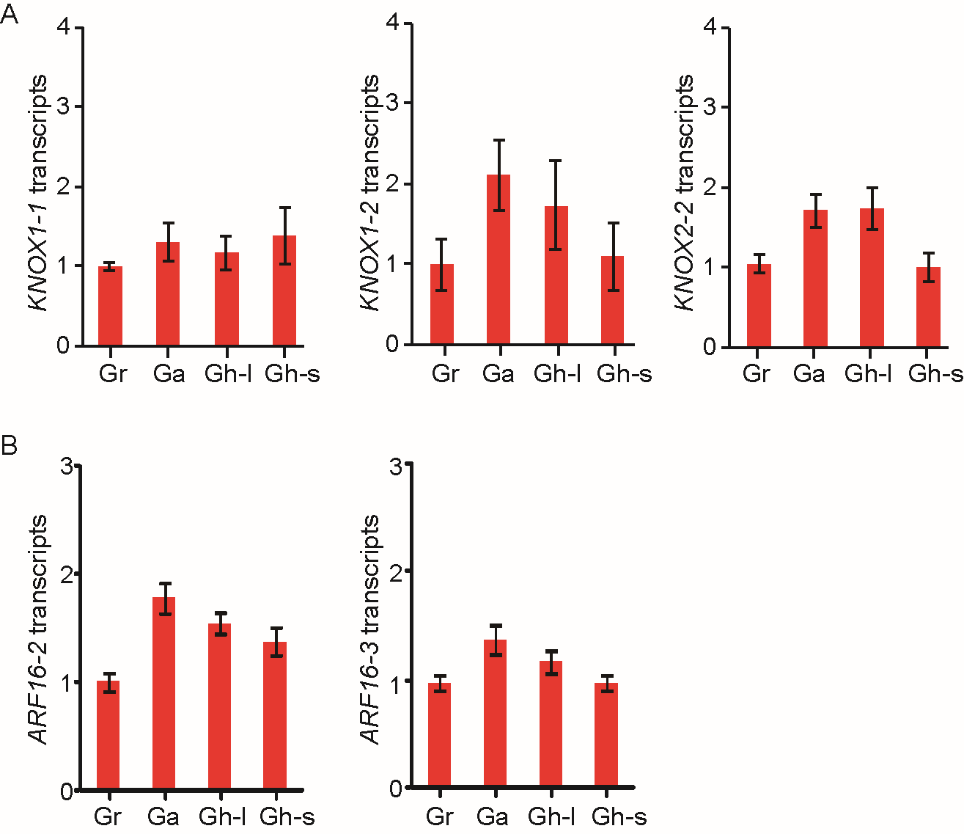
**

**Supplemental Figure S4** Analysis of Leaf Morphology and Transcriptional Level of *KNOX2-1* and *ARF16-1* from *G. raimondii* and *G. triobum*. (a) leaf phenotypes of *G. raimondii* and *G. triobum*. For each picture, leaves from left to right represent mature to young leaves. Bars = 4 cm. (b-d) Quantitative comparisons of leaf shapes of *G. raimondii* and *G. triobum* based on the leaf dissection index (perimeter^2^ / 4π × leaf area) in (b), the number of teeth / leaf perimeter in (c) and the tooth area / leaf area in (d). A total of 10 leaves of each phenotype were measured. Data presented are means ± SEs. (e) Expression level of the *KNOX2-1* gene in different leaves from *G. raimondii* and *G. triobum*. (f) Expression level of the *ARF16-1* gene in different leaves from *G. raimondii* and *G. triobum*. Each qRT-PCR experiment was performed in three biological replicates, and the error bars represent standard errors of the means from three independent experiments. Statistical significance was determined using one-way analysis of variance combined with Tukey’s test. *, *P<*0.05; ***, *P<*0.001.

**
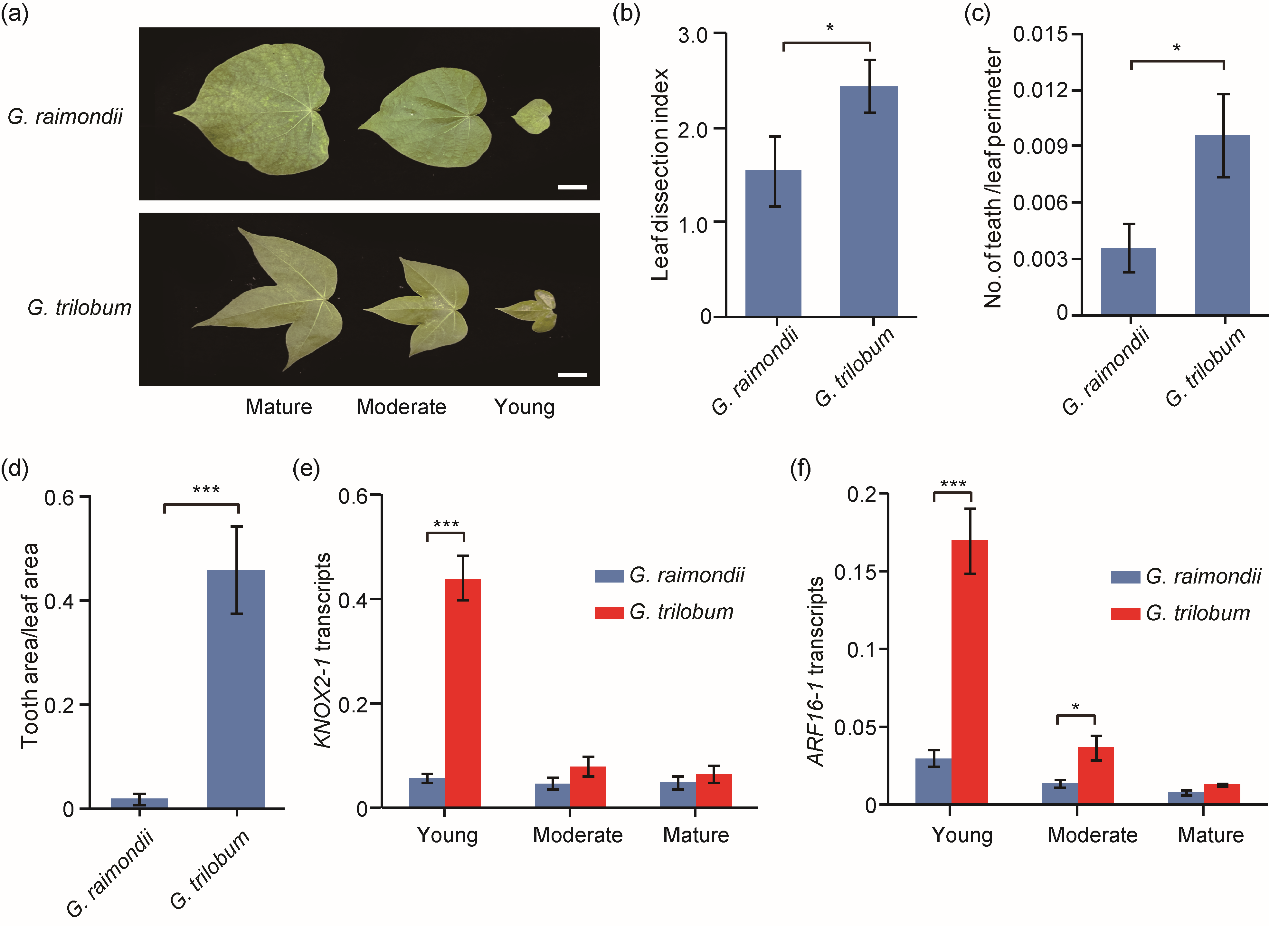
Supplemental Figure S5** A phylogenetic tree was constructed with MEGA 6.0 using the Maximum Likelihood (ML) method with 1000 bootstrap replicates based on a multiple alignment of the amino acid sequences of the Arabidopsis ARF proteins.


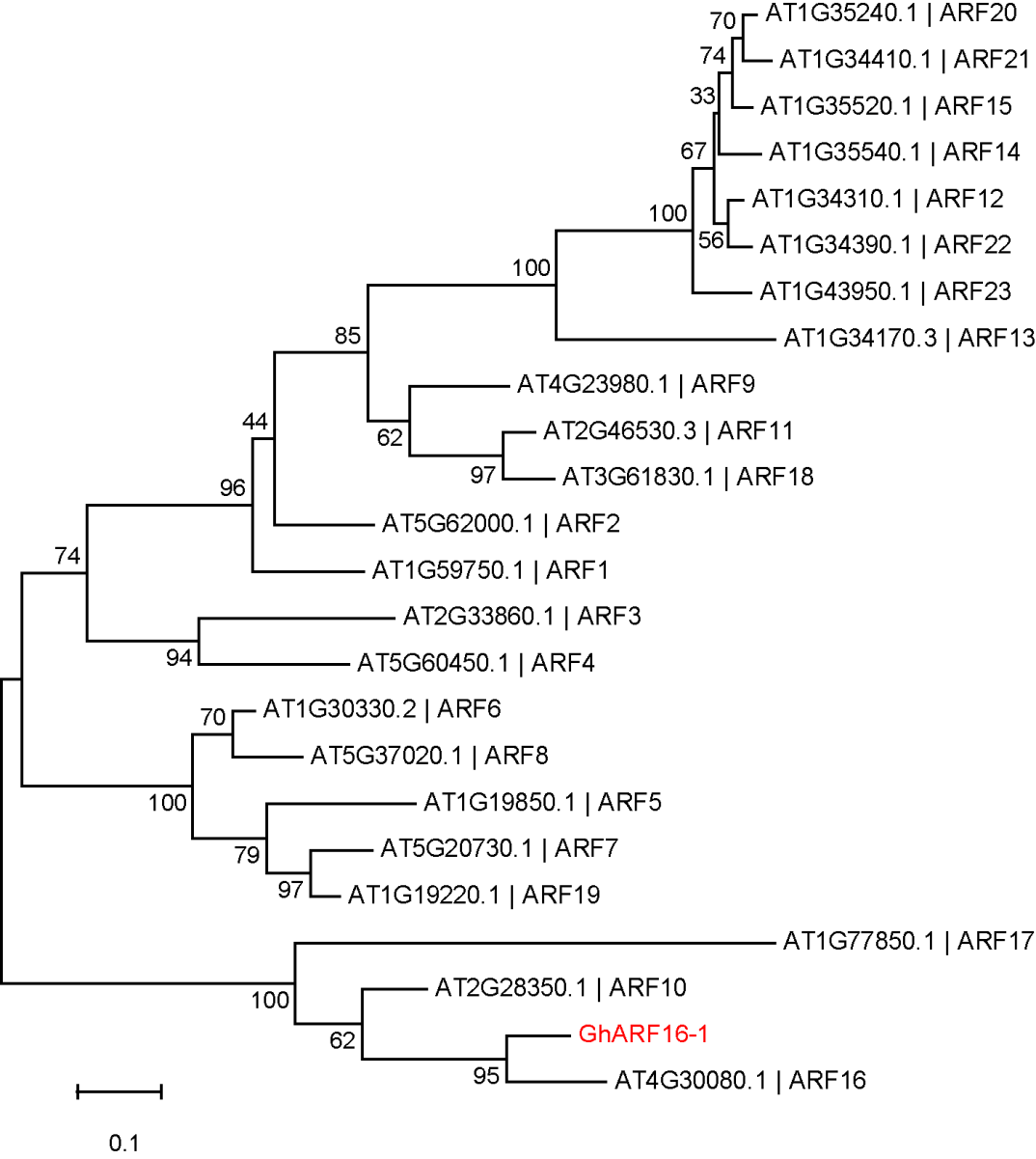


**Supplemental Figure S6** The Sequences of *GhARF16-1*, *GhmARF16-1* and miR160. *GhmARF16-1* was the modified *GhARF16-1* mRNA that harbor synonymous nucleotide substitutions in miR160 binding sites.

**
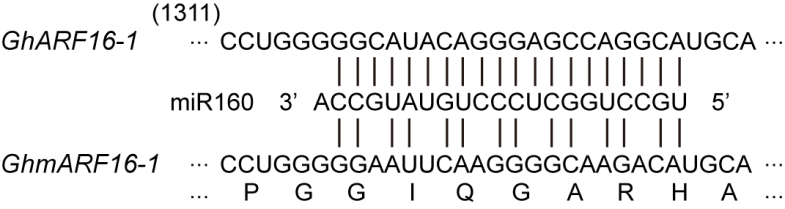
**

**Supplemental Figure S7** qRT-PCR Analysis of *GhARF16-1* mRNA Levels in Wild-Type, *GhARF16-1* RNAi and *pARF16::GhmARF16-1* Transgenic Plants. Each qRT-PCR experiment was performed in three biological replicates, and the error bars represent standard errors of the means from three independent experiments. Statistical significance in this figure was determined using one-way analysis of variance combined with Tukey’s test. ***, *P<*0.001. WT, wild-type.

**
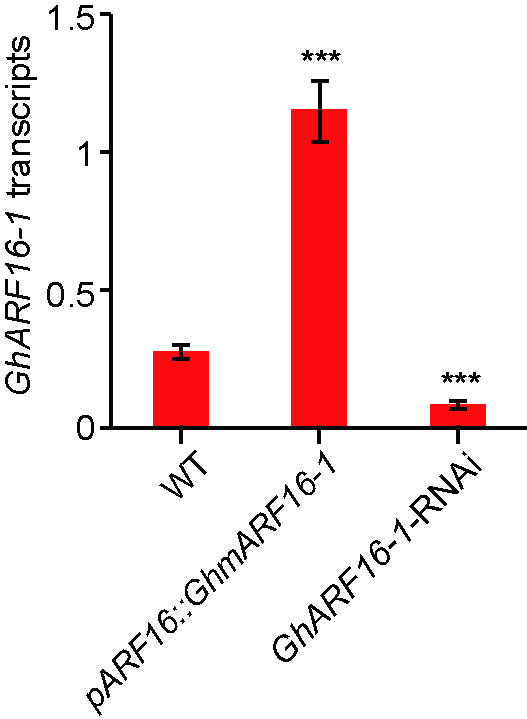
**

**Supplemental Figure S8** qRT-PCR Analysis of *GhKNOX2-1* mRNA Levels in Wild-Type, *GhKNOX2-1* RNAi and *35S::GhKNOX2-1* Transgenic Plants. Each qRT-PCR experiment was performed in three biological replicates, and the error bars represent standard errors of the means from three independent experiments. Statistical significance in this figure was determined using one-way analysis of variance combined with Tukey’s test. ***, *P<*0.001. WT, wild-type.

**
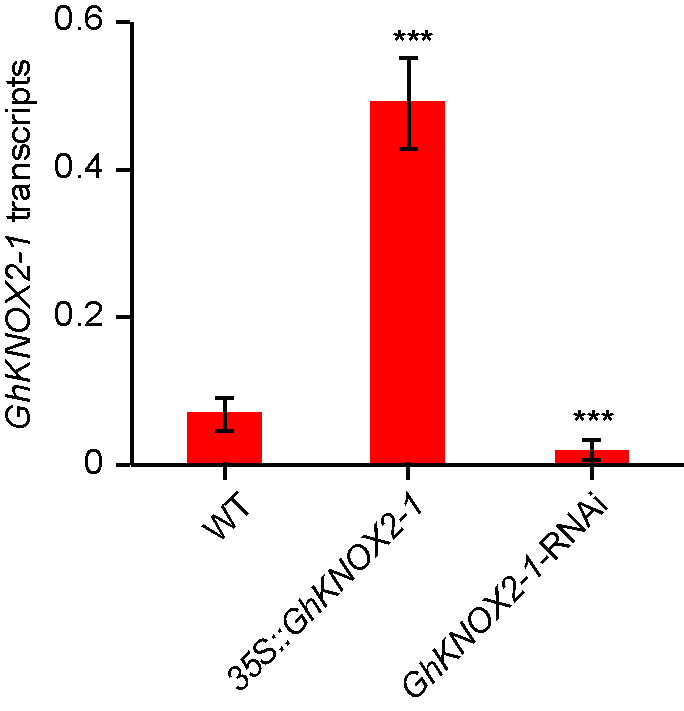
**

**Supplemental Figure S9** Subcellular Localization of *GhKNOX2-1* Using *N. benthamiana* Leaves. The nuclei were counterstained with DAPI. Merge, the merged images of mCherry (GhKNOX2-1-mCherry), DAPI and bright field. Bar = 50 µm.

**
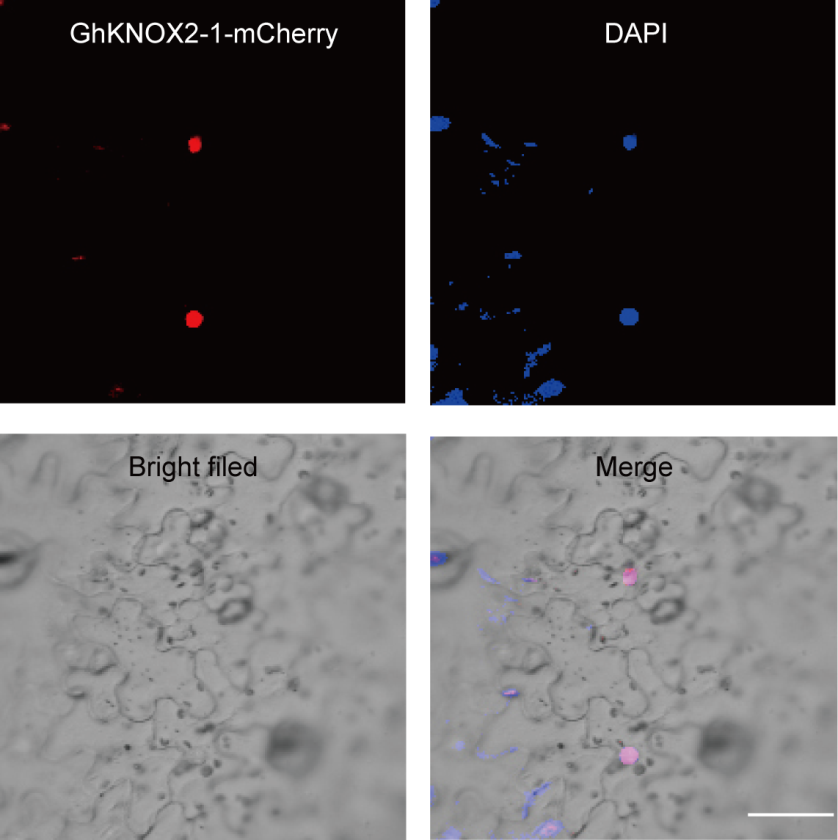
**

**Supplemental Figure S10** Transcriptional Ability of *GhKNOX2-1* in *Arabidopsis* Protoplasts. Left, schematic diagram of various constructs used in the transient expression assay. Right, transcriptional activity of *GhKNOX2-1*. The empty GAL4 DNA-binding domain (BD) and BD-VP16 vectors were used as negative and positive controls, respectively. Transcriptional activation assay was performed in three biological replicates, and the error bars represent standard errors of the means from three independent experiments.

**
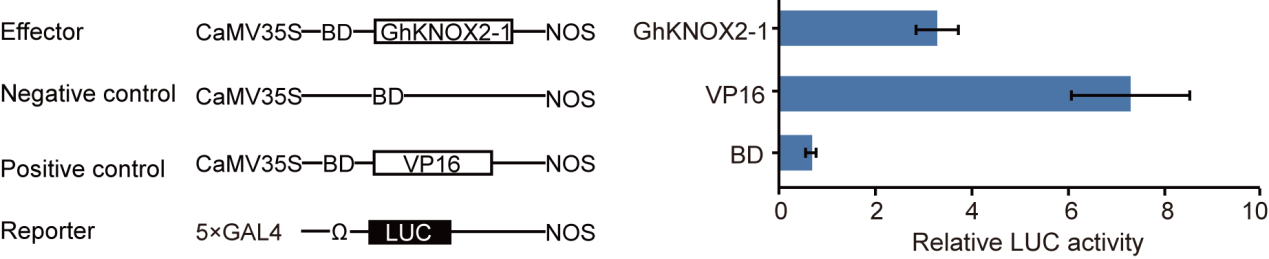
**

**Supplemental Figure S11** qRT-PCR Analysis of *GhARF16-1* mRNA Levels in *pARF16::GhmARF16-1* transgenic *Arabidopsis*. Each qRT-PCR experiment was performed in three biological replicates, and the error bars represent standard errors of the means from three independent experiments. DAG, days after germination.


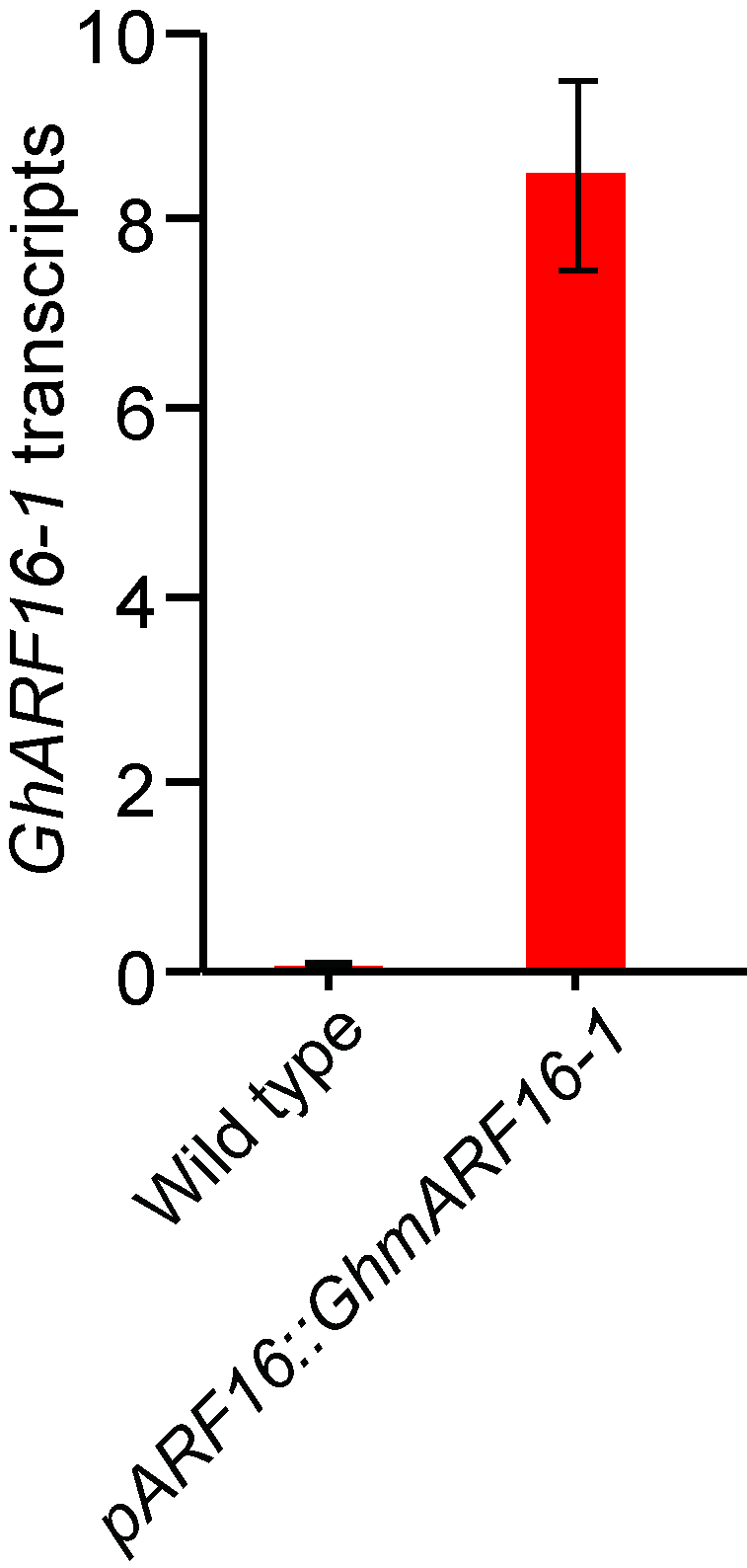


**Supplemental Figure S12** Scanning Electron Micrographs of the Leaf Epidermal Cells at the Base of Abaxial Sides of Mature Leaves from Wild-Type, *arf16* Mutant and *pARF16::GhmARF16-1* Transgenic Plants. Bars = 1 mm.

**
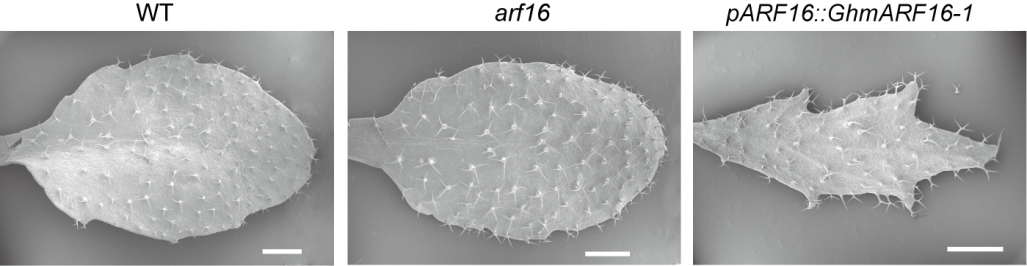
**

**Supplemental Figure S13** qRT-PCR Analysis of *GhKNOX2-1* mRNA Levels in *35::GhKNOX2-1* transgenic *Arabidopsis*. Each qRT-PCR experiment was performed in three biological replicates, and the error bars represent standard errors of the means from three independent experiments. DAG, days after germination.

**
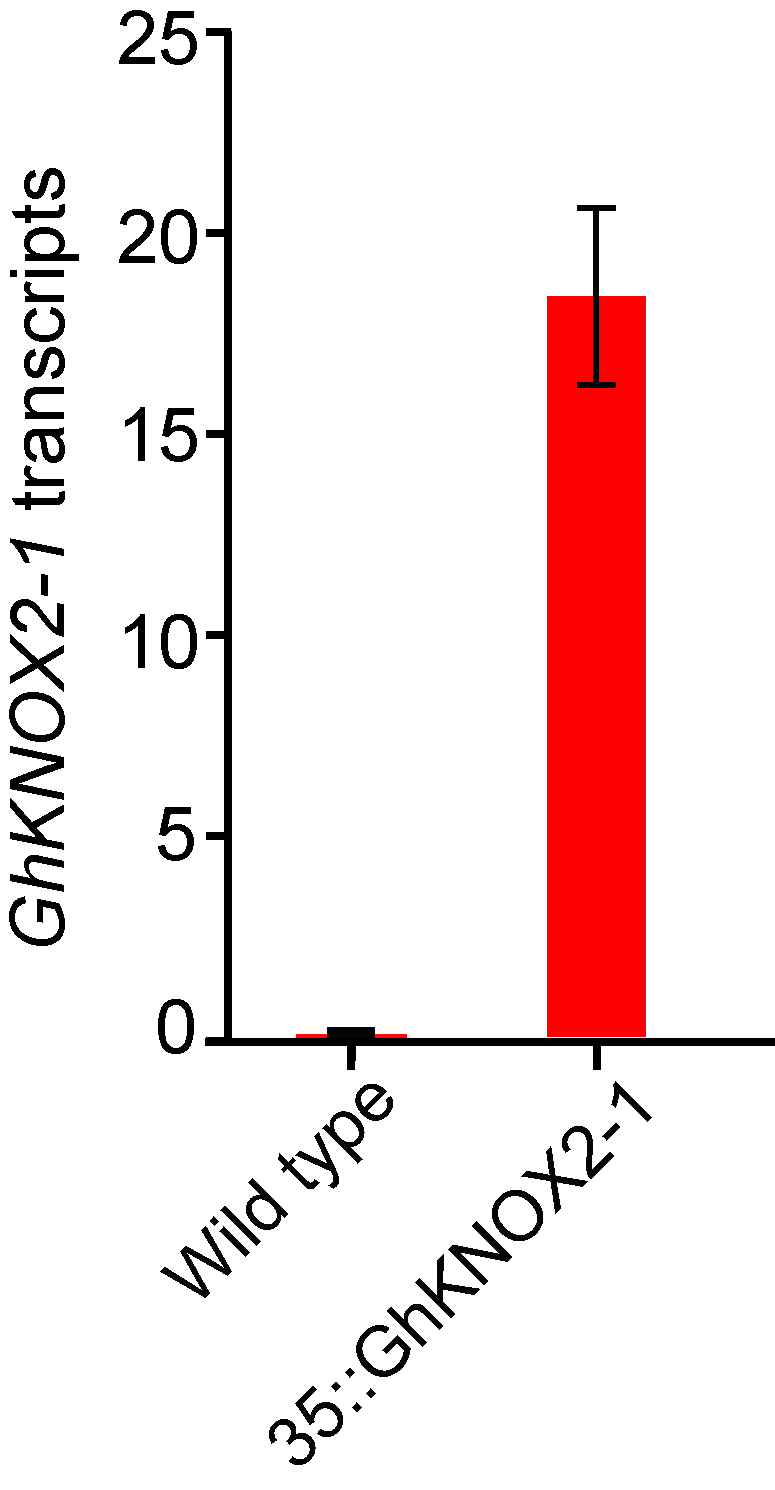
**

**Supplemental Figure S14** Scanning Electron Micrographs of the Leaf Epidermal Cells at the Base of Abaxial Sides of Mature Leaves from Wild-Type, *knat2* Mutant and *35S::GhKNOX2-1* Transgenic Plants. Bars = 1 mm.

**
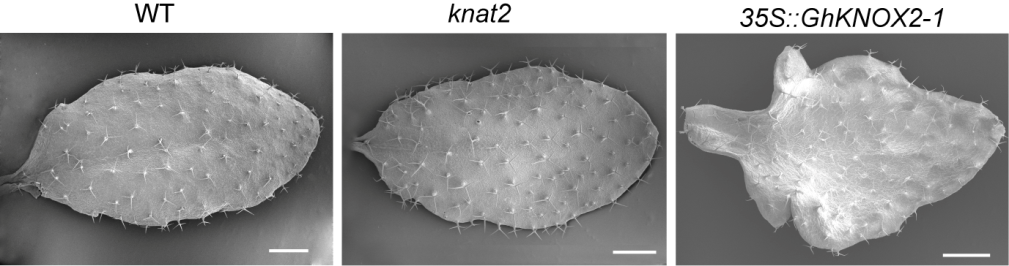
**

**Supplemental Figure S15** qRT-PCR Analysis of *GhKNOX2-1* mRNA Levels in Leaves from Individual Lines Shown in Figure 5A to 5D. Each qRT-PCR experiment was performed in three biological replicates, and the error bars represent standard errors of the means from three independent experiments. Statistical significance in this figure was determined using one-way analysis of variance combined with Tukey’s test. ***, *P<*0.001.

**
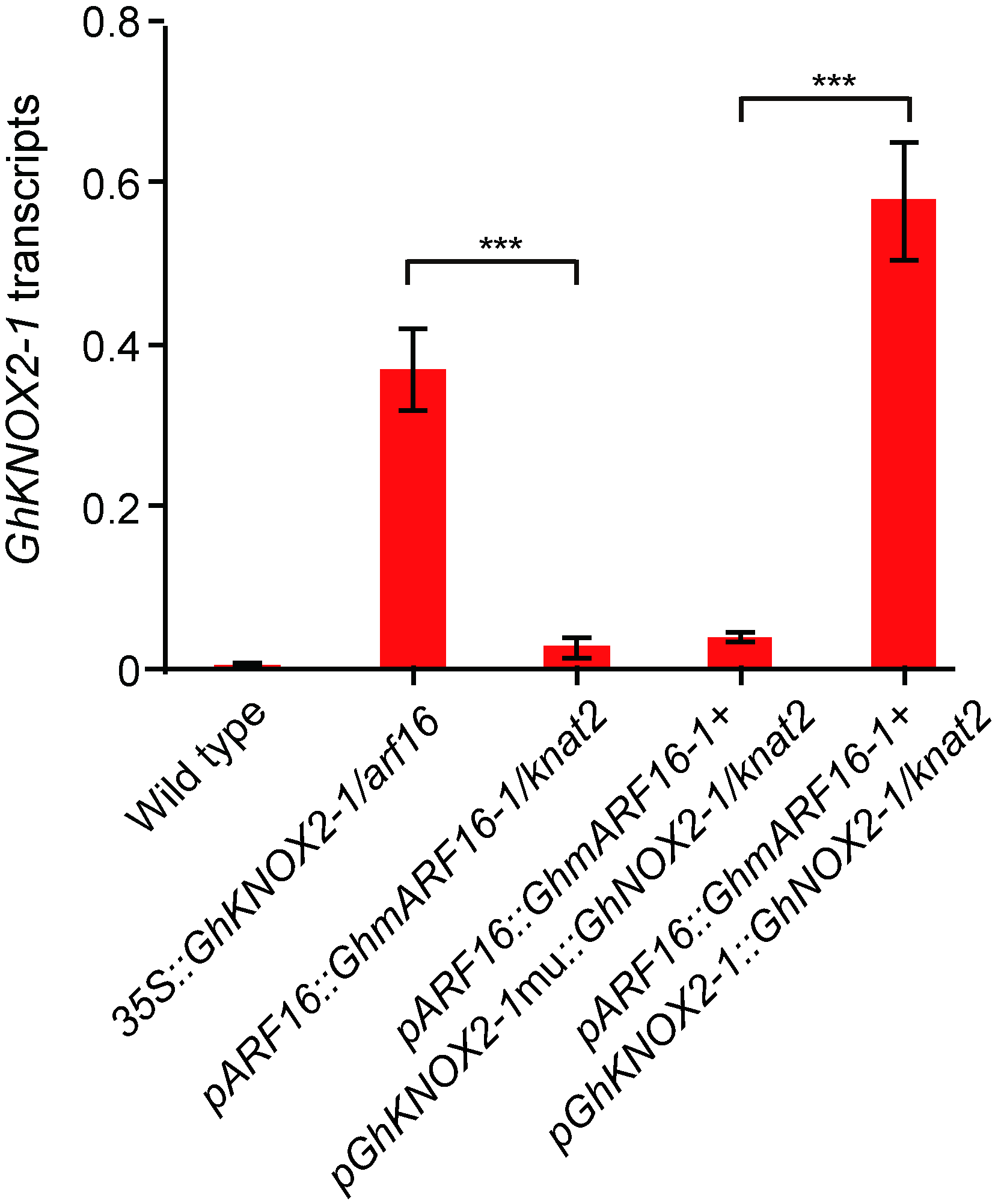
**

**Supplemental Table S1.** List of Genes Related to Leaf Shape Development in *Arabidopsis*.

| Gene Name | Accession No. | Reference |
| --- | --- | --- |
| *CUP-SHAPED COTYLEDON2* (*CUC*)  *CUP-SHAPED COTYLEDON3* (*CUC3*) | AT5G53950  AT1G76420 | Nikovicsetal, *et al*., 2006;  Hassonetal *et al*., 2011; |
| *KNOTTED1-like homeobox1* (*KNOX1*)  *KNOTTED1-like homeobox2* (*KNOX2*) | AT4G08150  AT1G70510 | Lincoln *et al*., 1994;  Pautot *et al*., 2001; Barkoulas *et al*., 2007; |
| *SHOOT MERISTEMLESS* (*STM*) | AT1G62360 | Spinelli *et al*., 2011; |
| *BLADE ON PETIOLE1* (*BOP1*) | AT3G57130 | Ha *et al*., 2003; Du *et al*., 2018; |
| *ASYMMETRIC LEAVES1* (*AS1*)  *ASYMMETRIC LEAVES2* (*AS2*) | AT2G37630  AT1G65620 | Jun *et al*., 2010;  Semiarti *et* *al*., 2001; Zgurski *et* *al*., 2005; |
| *DICER-like4* (*DCL4*) | AT5G20320 | Xie *et* *al*., 2005; |
| *AUXIN RESPONSE FACTOR3* (*ARF3*)  *AUXIN RESPONSE FACTOR4* (*ARF4*) | AT2G24765  AT5G60450 | Koyama *et* *al*., 2010;  Dkhar and Pareek, 2014; |
| *Arabidopsis phabulosa-1d* | AT2G34710 | McConnell *et* *al*., 1998; |
| *jagged and wavy* (*jaw*) | AT4G23713 | Palatnik *et* *al*., 2003; |

**Supplemental Table S2.** The promoter sequence of *GhARF16-1*.

AAGCTTACCTTAATTAATTGTCGAGTATTTATTTTTCACCTTAGATATAAAATAGTTTAAAAATTCATGATCAATATTTATCTCTTAATGAATCTACATAATGCAAAAAATTGATCGCCGTAACAAATACGTTGTAAGAAAAAAAAAACCCAACACTAATTGAAAAAATCACAAAAATTAGTTGTTGTTTGCTTTTTGGGGGGTTTGATTTGTTGGCTAAAATTTTGGTACCCTAAAAAAAGGGTCTAAAACAAGACCGACCAAGGTCTTAATCAAGTGCCGAATAATTTGGTTTTTTCATGCAACACTTGTAATCAAACAGATTAAATTGTCCTTAATCTCAATGTCACCATCATCTAATTTTACCAACTAATATTCCTTAATCCATGCATAGTATCTCGCTTTTAATTTTTGACCTCTAATAATTATCATTATATTAACACTTTCATCTATCTAATCACATTAACACTCTTTTTTTTTTTCCAATTCAACAAGTCTAAAATTAAATTTAATGCCCTGTTTAATTATAATTACTATAAATCCATACTCCATTAGTAATAACAACACCTTTTAATTTTTAATTATATATATCATTCCATTAAATAATAACCCTAATTTCAAGGGTAAATTTGTAATTTCATATTTTGATAAAAAAAAGTGTTTTTCTCTTTGGAAAAAAATGTTAAACCCTAATCCGAGTACTAAGGATAAAATCTTGTCGTTCGGATCTTAGGATCAAGAAAGACAATAACAAACACTGAAAAGAAGAGTCAAAGTGTTGACCAGTTAATTAAAAAGATAATTAATATAAATAGATTAATTACTTAAAAAAACGAGCCTTTAGATTTTGTAGTTTATGTAAGTTAAATAAAAATAAGATAAAGAAAAAGTTAAAATACACTTGGCACTTATTTAATGGACTAAATAAAAATTAAAAAAAGAGAGAAAATAGGTGGAAAAATATTTTTATTTTTCTTTGATAGAAAAAAAAATGTATTGATTTTTATAATAATAAAAAGAATTGAAAATATTTTTCAATGGCAAGTGGCATACATGATAATATTTTGGGAAATAAATTGTTTTTGCTTTATTTCCTTAAATAATAACTAAATAATCTATTATAAATTACTCACGAAAAAATGATAATTTTTCATATCTTTGGAAAAAAATATTGAATAGCTAATATATTTTATAATTTTTTAAACAAATTAATATTATTTATATAAATTAAAAAATAAATAAATTAACCATGGTTAGTCCCGAAGAGTTAGGGCCTCGGACAAAATGAAAACTTTAACTGTAGTCAGCCTTGATTTCAGAGTTGCATGAAAATCGTGATATCTTCCAGCAAAATATCGTGATATTTAAAATCAACATAAAAAATAATTTTTAAAAAAATTAAAACTTTTTTTGGGGATTATCTAAATATTTTAATTTAGCAGAGAGAGAGATTTATTAAATAATTATAAAATTAAATTCTCTGATTAATTTTTATTAGTTGGATGTTTCCTTTTTCTGATTTGTAGGGGATATGTTCCCACTTGCTTCCTTGCCGCGTGGTAAACACTCCTTGTCAAAATAAAGCCACAACTGTCCAGGTCTGAAACAATAAAATAAAATAAATTATATTTTAAAACTTTATAATTAGAAAAATATAATTTTTTACTTGATATAAATAAAAAACTTATAAATCCTTTCCCGAATCGAACCGGAACCGGACCGAAGTAAATACTGACAAGGGCTTTTAACAGAGAGTCCCTGGTCGGCAAATAACACAAAATCTTTCACTGTTAAAATCTCTCATCTCATCTTCTCTTCTCATCTTCTTTAAGGATCC

**Supplemental Table S3.** The promoter sequence of *GhKNOX2-1*. TGTCTC, marked by red, is the ARF binding site.

TATCTCTCACTATCTTATCTTTCTTTTCGTTTCCCCCTCTAGTTTGTTCCCTCAAGGGCTTTGCTATATAACAACACAACCCATTTTCTCTCTCTTCTCTTTCCTACGCAATCTATTTTTTTGCCCTAAAGATTGTTAATATCTCTACAACTCTCCCGAGATTTATGCTACAAACAACAGGCTTTTCATAGGAACTCCTAAATAGTGATAGCAGCAGCAGCAGGAGGTAGTGGAGTTTGAGAAAAATGTGGGATTTTACTCTCTGGGGCTAACCTGGCAAAGCCTGCTCAGATTTCCTTTGGAAAATGGGAGTTTGTTTGTTGATCTTCCTAGTCATTAGCAGAGAGCATTAATTCTCACTTTCTATTACAAAATCCTCTC**TGTCTC**TCGAGCTTCTTTTGCACTTGTTTGGAAGAAAAGAAAGACAGAAAAAAGGAAGGGGAAGGGGGTTTGGTGTGTTTGCAGAACGCACGCGGGCTATGCAGAAAGAAAGCAAGGCATTGAAAGAACTTCAAATCTCATGGCTCTACTAGTAAGCAGGTGTGGGGGATATAGTGGGGGTGTGTTTGAGGCAGTACTGGTGGCTGACTGCCTGTGGCTGGGATATGCAAAGCTTTACCTTCCCGATGAAAACTCTCCCCTCTTTTCTTGCACCGCTAACTGATGCACTGCCATTTTACTGGCAAACTACAAAGAGAAAAAAACAGATAAGTGAGGGAGATTAGACGTTTATAATTTGTTATCTTATCGTTTACTCCATATATTACAAAAAGGAAACCGATAAATAATAATAATACAACAATAGAGGGATGAAACGATCAAATCGAGGAAGACTGTAAGGCAAACTAGTGGGAAAAGGTTTTATGGGCCACGAAATCATCAATTCATTAAATTCCTCTTCTCCTTCTCCTTCTCCTTCTCCTTCTCCTTCTCCTTCTCCTTCTCCTTCTCCTTTTTCTACTCTCTTTTAATTTATGTAAATGTTGTGTTATGTAGTGTACGTACTGTTTTCAGTTTTCCTTTTTGTTTTTTAACAATTTTATTATAGATTTTGTTATTATTTTTAATTCTTAAATAAAAAGATAATGCGCTTCAACACACTCGAATTTACGTTCTCCTTCATTAGTTACAGTAATTAAGATTCAATTGACTAAATTCAAATGTTATTAGAAGTACGAAATGATATTCAGTTTAAGGATTTTATATTTATTATGGTAATTTAATATTCTAGAACTGCATTCCATTATTTGGATTTTGAAAATTATCGCATTTACATTTCCAACTATCTTTTGGTAATATACGTGCTAATTTCAATATTACATATTTCTTAAGATGCATTTGATTCGTGGAAGGTAACATTTTATCCTATAATTTCATATTTCGAAATAATATTATATTGTTTAGATTGTTAATATTCTAACATCCCGTAATTTTACATTCTTAAAGAGTGTCAAAATATCATAATATGAGGATAATATTAATATCACTTATTATTAGGTTA

**Supplemental Table S4.** Analysis of *G. hirsutum* *KNOX* Gene Family and Its Orthologs in AA and DD Cotton Genomes.

| Gene ID | Length of amino acid | Number of intron |  | Gene name | Orthologous | Type |
| --- | --- | --- | --- | --- | --- | --- |
| CotAD_36041 | 350 | 5 |  | GhKNOX2-1 | Cotton_A_09092 | Class I |
| CotAD_25269 | 234 | 1 |  | GhKNOX1-1 | Cotton_A_25268 | Class I |
| CotAD_33195 | 384 | 3 |  | GhSTM-1A | Cotton_A_08468 | Class I |
| CotAD_19295 | 296 | 4 |  | GhSTM-3A | Cotton_A_33973 | Class I |
| CotAD_61993 | 290 | 4 |  | GhKNOX5-1A | Cotton_A_08384 | Class I |
| CotAD_72428 | 308 | 4 |  | GhKNOX6 | Cotton_A_33669 | Class I |
| CotAD_32400 | 369 | 6 |  | GhKNOX1-3A | Cotton_A_25269 | Class I |
| CotAD_36715 | 314 | 4 |  | GhKNOX2-1 | Gorai.008G219640.1 | Class I |
| CotAD_53081 | 310 | 4 |  | GhKNOX1-3D | Gorai.008G394000.1 | Class I |
| CotAD_00861 | 350 | 3 |  | GhSTM-1D | Gorai.008G312100.1 | Class I |
| CotAD_32314 | 357 | 3 |  | GhSTM-3D | Gorai.008G351040.1 | Class I |
| CotAD_38890 | 290 | 4 |  | GhKNOX5-1D | Gorai.008G044330.1 | Class I |
| CotAD_06890 | 499 | 5 |  | GhKNOX3-1A | Gorai.012G090920.1 | Class II |
| CotAD_40979 | 425 | 4 |  | GhKNOX4-1A | Cotton_A_01196 | Class II |
| CotAD_50793 | 295 | 4 |  | GhKNOX5-2 | Cotton_A_11503 | Class II |
| CotAD_45200 | 299 | 4 |  | GhKNOX7-1A | Cotton_A_28415 | Class II |
| CotAD_28024 | 311 | 4 |  | GhKNOX7-2A | Cotton_A_14429 | Class II |
| CotAD_25407 | 299 | 4 |  | GhKNOX7-3A | Cotton_A_09961 | Class II |
| CotAD_10962 | 449 | 5 |  | GhKNOX3-1D | Gorai.005G267030.1 | Class II |
| CotAD_72711 | 433 | 4 |  | GhKNOX3-2 | Gorai.012G395350.1 | Class II |
| CotAD_10451 | 315 | 4 |  | GhKNOX3-3 | Gorai.005G153140.1 | Class II |
| CotAD_00115 | 425 | 4 |  | GhKNOX4-2D | Gorai.005G370970.1 | Class II |
| CotAD_60713 | 308 | 4 |  | GhKNOX4-3 | NA | Class II |
| CotAD_20435 | 299 | 4 |  | GhKNOX7-1D | Gorai.008G010800.1 | Class II |
| CotAD_37413 | 311 | 4 |  | GhKNOX7-2D | Gorai.006G169620.1 | Class II |
| CotAD_31075 | 300 | 4 |  | GhKNOX7-3D | Gorai.007G171450.1 | Class II |

**Supplemental Table S5.** Analysis of *G. hirsutum* *ARF* Gene Family and Its Orthologs in AA and DD Cotton Genomes.

| Gene ID | Length of amino acid | Number of intron | Gene name | Orthologous |
| --- | --- | --- | --- | --- |
| CotAD_08070 | 674 | 2 | GhARF16-1 | Cotton_A_06107 |
| CotAD_76501 | 685 | 2 | GhARF16-2 | Cotton_A_23397 |
| CotAD_66018 | 708 | 2 | GhARF16-3 | Cotton_A_24047 |
| CotAD_44201 | 708 | 2 | GhARF16-3D | Gorai.005G063970.1 |
| CotAD_61329 | 674 | 2 | GhARF16-1D | Gorai.005G084560.1 |

**Supplemental Table S6.** A List of Primers Used in This Study.

| **Name** | **Sequence 5’-3’** | **Experiment** |
| --- | --- | --- |
| GhKNOX2-1-BamHI-F | CGCGGATCCATGGAAGAATATAATCGAGTAAAC | pCAM2300:: *GhKNOX2*-mCherry |
| GhKNOX2-1-EcoRI–R | TCGGAATTCAGATGGGCCCGGACTATAAGGG |  |
| GhARF16-1-KpnI-F | CGGGGTACCATGATAACAGTCATGGATTCTAG | pCAMBIA1305:: *GhARF16-1*-GFP |
| GhARF16-1-XbaI-R | CGCTCTAGATCTTTCTAAGTTGTCACTGCTTG |  |
| GhARF16-1-pTRL-F | TTCACTAGTATGATAACAGTCATGGATTC | 35S::BD-  *GhARF16-1* |
| GhARF16-1-pTRL-R | GACGAATTCCTATCTTTCTAAGTTGTCAC |  |
| GhKNOX2-1-pTRL-F | TTCACTAGTATGGAAGAATATAATCGAG | 35S::BD-  *GhKNOX2-1* |
| GhKNOX2-1-pTRL-R | GACGAATTCAGATGGGCCCGGACTATAA |  |
| GhKNOX2-1-pro-GUS-F | CGCGTCGACACGGGATGTTGGAATGTTGA | GUS Staining |
| GhKNOX-pro2-1-GUS-R | CGCGGATCCACTCTCTGGGGCTAACCTGG |  |
| GhKNOX-pro2-1-H1Y-F | CGCGAGCTCACGGGATGTTGGAATGTTGA | Yeast one hybrid |
| GhKNOX-pro2-1- H1Y-R | CGCGGTACCACTCTCTGGGGCTAACCTGG |  |
| GhARF16-pGADT7-F | AGAGTGGCCATTATGGCCCATGATAACAGTCATGGATTC | Yeast one hybrid |
| GhARF16-pGADT7-R | GCGGCCGACATGTTTTTTCCCCTATCTTTCTAAGTTGTCAC |  |
| KNOX2-1-F | ATCATTGCTCATCCTCACTGCT | qRT-PCR |
| KNOX2-1-R | CTGGTTTTGGTTCCTCCATTACT | qRT-PCR |
| KNOX7-3-F | GGTTTTGGTCCCTTGATTCC | qRT-PCR |
| KNOX7-3-R | GTGACAGATTGAGAGTTGCTAT | qRT-PCR |
| KNOX1-3-F | CGGGAGGAACTAACGAGACCA | qRT-PCR |
| KNOX1-3-R | TCCAAAGGAATCCAAGCAAA | qRT-PCR |
| STM-1-F | CCTCACGGTCTCATCTTCAAAT | qRT-PCR |
| STM-1-R | TGTAATGTCTACTCCACCAATCC | qRT-PCR |
| KNOX1-1-F | TCAAGACAATAGTGGCGGAGAA | qRT-PCR |
| KNOX1-1-R | GTTTCCAATGGCGTTTCCTT | qRT-PCR |
| STM-2-F | ACAGTTGCTGGATTGGTGGA | qRT-PCR |
| STM-2-R | GAAGGGATTGCCCAAAACAT | qRT-PCR |
| KNOX6-F | ATGTTAGTGTTGGGGAGGATGA | qRT-PCR |
| KNOX6-R | AGCCAATGCCATCTTATCACC | qRT-PCR |
| ARF16-1-F | GTGAAGTGTGTTAGCCCATGG | qRT-PCR |
| ARF16-1-R | CGTGAAGACTTGGCTATGACC | qRT-PCR |
| ARF16-2-F | GTTATTCTCCGTTTTTGAGGG | qRT-PCR |
| ARF16-2-R | GAGAAAGGATTAAGATTAATAG | qRT-PCR |
| ARF16-3-F | GCTCAAATGGTAATTTGATGG | qRT-PCR |
| ARF16-3-R | AATCCGAGTGCTGCGGCAGTC | qRT-PCR |
| UBQ7-F | GGCATTCCACCTGACCAACAA | qRT-PCR |
| UBQ7-R | CCGCATTAGGGCACTCTTTTC | qRT-PCR |
